# Miro1 protects against brain injury after CPR in rats by enhancing the effect of BMSCs on mitochondrial homeostasis

**DOI:** 10.1186/s13287-025-04724-5

**Published:** 2025-10-28

**Authors:** Xuyuan Ma, Maozheng Shen, JiaYu Hu, Jie Zhu, Zhen Wang, Qian Zhou, MingYue Zeng, Mengda Xu, YanLiang Qu, Xiang Zhou

**Affiliations:** 1 Base of Central Theater Command of People’s Liberation Army, Hubei University of Medicine, Wuhan, China; 2 Department of Anesthesiology, Zhongnan Hospital of Wuhan University, Wuhan, China; 3 Department of Anesthesiology, General hospital of central theater command of PLA, Wuhan, China; 4 ChinaPLA Navy No.971 Hospital, Qingdao, China

**Keywords:** Bone marrow-derived mesenchymal stem cells, Cardiopulmonary resuscitation, Mitochondria, Miro1

## Abstract

**Background:**

Mitochondrial dyshomeostasis plays an important role in neuronal damage after cerebral ischemia-reperfusion, and Miro1 is a core protein that regulates mitochondrial homeostasis. In this study, we aimed to investigate the neuroprotective effects of bone marrow-derived mesenchymal stem cells (BMSCs) via mitochondrial homeostasis in rats after cardiac arrest (CA), and to clarify the role that the protein Miro1 plays in this protective efficacy.

**Methods:**

The study compared the effects of BMSCs in which Miro1 was overexpressed BMSCs (BMSCs-miro^hi^), knocked down (BMSCs-miro^lo^), and unmodified BMSCs on mitochondrial homeostasis in hippocampal neurons to evaluate their neuroprotective effects of these cells in a rat model of global cerebral ischemia-reperfusion injury. Rats underwent CA modeling for 5 min and received cardiopulmonary resuscitation (CPR). Two hours after the restoration of spontaneous circulation, 1 mL of PBS or 1 mL containing 1 × 10^6^ BMSCs (normal, miro^hi^, or miro^lo^) were injected via the femoral vein. The neurological function of rats was assessed based on Neurological Disability Score (NDS) values. Brain histopathological examination was conducted to evaluate brain injury by measuring oxidative stress levels and the apoptosis rate of hippocampal neurons. Immunoblotting and transmission electron microscopy were applied to detect the expression of mitophagy-related proteins in hippocampal neurons. Immunofluorescence was used to track the mitochondria in BMSCs and observe mitochondrial transfer. Additionally, the membrane potential level, oxidative stress level, and ATP content of mitochondria in hippocampal neurons were measured to assess the impact of transplanted BMSCs on mitochondrial quality in these hippocampal neurons.

**Results:**

Immunofluorescence staining revealed the presence of mitochondria from MitoTracker-labeled BMSCs in rat hippocampal neurons post-CPR. Additionally, the fluorescence intensity of TOMM20 was notably increased following the transplantation of BMSCs. Through immunoblotting experiments, we identified that BMSCs amplified the post-CPR protein expression of LC3, p62, PINK1 and parkin in hippocampal neurons. The number of autophagosomes significantly increased in hippocampal neurons following BMSC transplantation, as observed through transmission electron microscopy. Flow cytometry, Hematoxylin and Eosin (HE) staining, and NDS scoring indicated that BMSCs effectively reduced reactive oxygen species accumulation in hippocampal neurons and mitochondria after CPR. Furthermore, they restored mitochondrial membrane potential and ATP levels in the hippocampus while decreasing apoptosis, ultimately contributing to the restoration of neurological function. Additionally, unlike BMSCs-miro^lo^, BMSCs-miro^hi^ were able to significantly enhance the efficiency of BMSC-mediated mitochondrial transfer and enhance mitophagy. This amplification, in turn, was found to bolster the protective impact of BMSCs on hippocampal neurons during CPR, thereby contributing to the restoration of rat neurological function.

**Conclusions:**

These analyses revealed that BMSC transplantation has a dual protective effect by facilitating healthy mitochondrial transfer and promoting the autophagic degradation of damaged mitochondria, effectively enhancing hippocampal neuronal mitochondrial function following CA while reducing neuronal apoptosis, restoring neuronal function, and alleviating neuropathological damage. Moreover, Miro1 can enhance the efficiency of mitochondrial transfer and promote BMSC-mediated mitophagy induction, thereby optimizing the therapeutic effect of BMSCs.

**Supplementary Information:**

The online version contains supplementary material available at 10.1186/s13287-025-04724-5.

## Introduction

Cardiac arrest (CA) poses a persistent threat to human well-being due to its high incidence rate and mortality [1]. The prompt initiation of high-quality cardiopulmonary resuscitation (CPR) by professionals is essential for saving individuals experiencing CA [2]. With advancements in medicine and the broad dissemination of emergency medical knowledge, the rates of CPR initiation and success have markedly improved. Nevertheless, only 20% to 40% of patients achieve restoration of spontaneous circulation (ROSC) [3]. Most deaths following ROSC are attributed to brain damage [4]. ROSC cerebral anoxia syndrome, which is characterized by injury caused by global cerebral ischemia/reperfusion (I/R), has an unfavorable prognosis. Transient global ischemia prompted by CA can lead to the targeted demise of delicate neurons, including hippocampal neurons [5]. Reperfusion injury after CPR has been identified as the predominant contributor to mortality in 68% of out-of-hospital CA cases and 23% of in-hospital CA cases due to brain damage [6]. Even in instances where ROSC is successfully achieved following CPR, individuals may experience a spectrum of neurological sequelae, consequently influencing their future quality of life.

Prior studies in rat model systems have revealed that the apoptosis and necrosis of hippocampal neurons are fundamental mechanisms contributing to neurological damage post-CPR. Mitochondria are particularly susceptible organelles during hypoxia [7]. Following CPR, damaged neuronal mitochondria [8]. These pathological alterations culminate in compromised mitochondrial function and the activation of the mitochondrial-mediated intrinsic apoptotic pathway, ultimately inducing significant neuronal apoptosis within the brain [9]. Therefore, enhancing neuronal mitochondrial function and mitigating neuronal apoptosis after CPR are pivotal strategies for bolstering post-CPR neuroprotection [10].

Mesenchymal stem cell (MSC) transplantation is an evolving protective strategy that can be employed following CPR and associated global cerebral ischemia/reperfusion in rats [11]. Bone marrow MSCs (BMSCs) are extensively used for the treatment of diverse degenerative conditions, including cardiovascular diseases and neurological complications due to their capacity for pluripotent differentiation and efficient preparation in vitro [12]. Recent research has verified that when neurons are exposed to stress, injury, ischemia, or hypoxia, local microenvironmental cellular damage can facilitate the one-way transmission of healthy mitochondria from various MSCs, including BMSCs, to damaged neurons [13]. Moreover, MSCs can induce mitophagy, raise mitochondrial membrane potential (MMP), decrease intracellular ROS levels, and enhance the viability and quality of target cell mitochondria, thereby improving cellular outcomes [14]. Nevertheless, the efficacy of conventional BMSC-mediated mitochondrial transfer remains suboptimal, and the enhancement of mitophagy intensity is constrained. As the pivotal protein for the process of mitochondrial transfer, Miro1 effectively controls the efficacy of intercellular mitochondrial transfer and governs the microtubule-assisted transport of mitochondria along axons and dendrites, thereby facilitating long-range mitochondrial movement and meeting diverse cellular metabolic demands [15]. Miro1 also plays a critical role in the regulation of mitophagy [16]. Augmenting the expression of Miro1 can substantially improve the efficiency with which impaired neuronal mitochondria are cleared, thereby enhancing the intracellular environment [17]. Therefore, in order to further optimize the efficacy, in this study, we used lentiviral vectors to induce Miro1 overexpression in BMSCs, and then detected the neuroprotective effects of these virus-transfected BMSCs on brain injury and neurological deficits after CPR in rats. These BMSCs were ultimately found to have neuroprotective effects on CA-induced global cerebral ischemia through mitochondrial transfer and mitophagy. Moreover, Miro1 was able to improve the efficiency of mitochondrial transfer, strengthen the intensity of mitophagy, and enhance the neuroprotective effect of BMSCs on CA-induced global cerebral ischemia.

## Materials and methods

### Animals

**Fig. 1:**
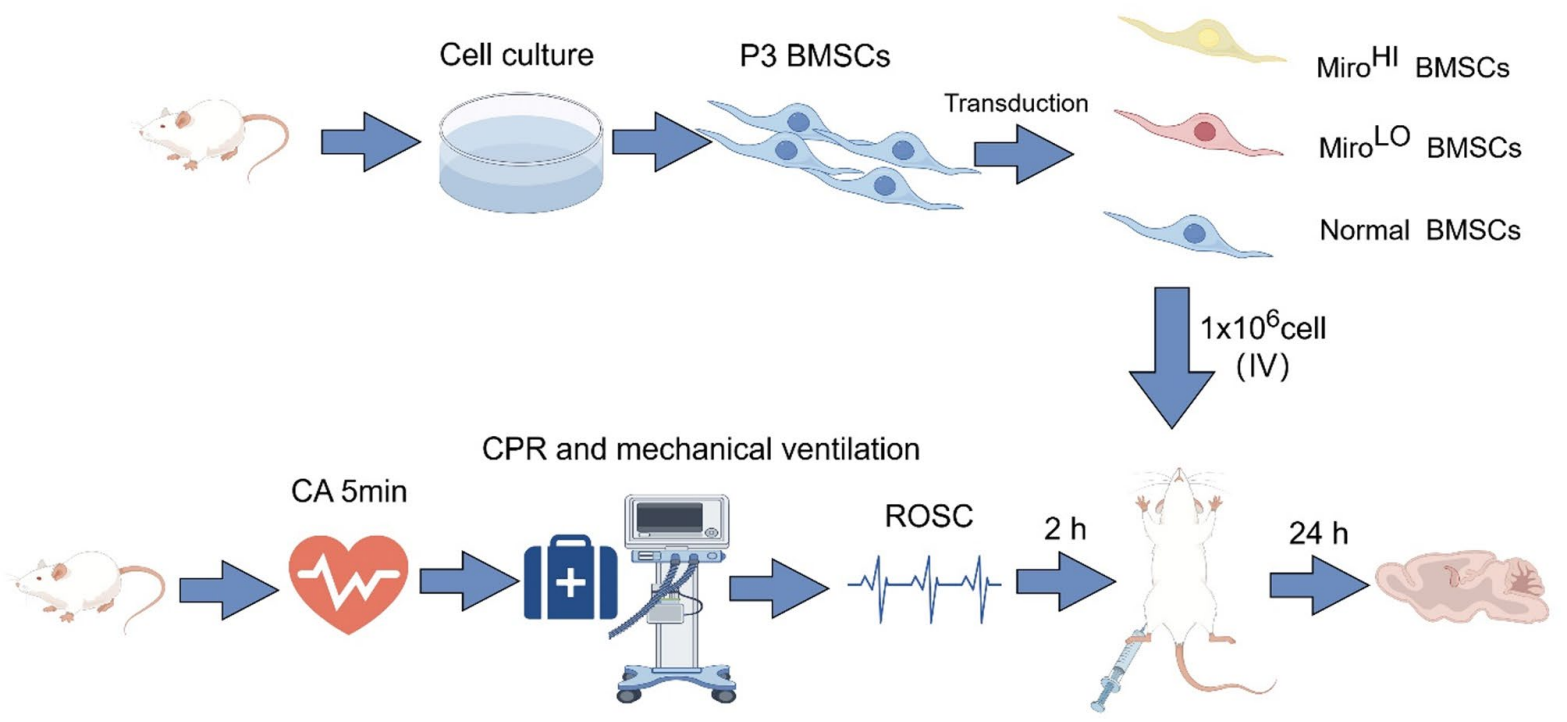
A schematic overview of the experimental approach

The work has been reported in line with the ARRIVE guidelines 2.0. Immature male Sprague-Dawley (SD) rats (4 to 5 weeks old, 100–150 g) and adult naive male SD rats (10 weeks old, 280–330 g) of SPF (specific pathogen-free) grade were obtained from the Hunan Silaikejingda Experimental Animal Company Ltd. (NO.430727231102711675, Changsha, China). The rats were housed in controlled conditions with temperatures ranging from 22 to 25 °C, humidity between 60% and 80%, and a 12-h light-dark cycle. The rats received proper care as per NIH guidelines. The experimental procedures for BMSC therapy are detailed in a flowchart (Fig. 1).

The study was divided into two parts. In the first, 60 rats were randomly assigned to the following three groups using a computer-based random number method (*n* = 20/group): a sham group in which rats underwent surgery without CA/CPR, a CPR-vehicle group in which rats were given 1 mL phosphate-buffered saline (PBS) via the femoral vein 2 h after ROSC, and a CPR-BMSCs group (*n* = 20) in which 1 × 10^6^ BMSCs in 1 mL were administered through the femoral vein 2 h after ROSC. In the second part of the study, 60 rats were randomly assigned to the following three groups as above (*n* = 20/group): BMSCs, BMSCs-miro^hi^, and BMSCs-miro^lo^ groups in which rats were respectively administered 1 × 10^6^ BMSCs, BMSCs overexpressing Miro1 (BMSCs-miro^hi^), or BMSCs in which Miro1 had been knocked down (BMSCs-miro^lo^) in 1 mL via the femoral vein 2 h after ROSC. Every effort was made to minimize animal discomfort and usage during the study. At 24 h after ROSC, 10 rats were euthanized under deep anesthesia to obtain their brains, 10 rats euthanized after assessing neurological function within a week.

### Model of asphyxial CA

The protocol for establishing the rat model of asphyxial CA was performed in accordance with the modified Utstein-style guidelines [18] and as described previously [9]. The rats received general anesthesia via an intraperitoneal injection of pentobarbital sodium (40 mg/kg). Anaesthesia was maintained by giving additional pentobarbital (10 mg/kg) as needed. After the rat’s righting reflex has diminished, the right femoral artery and vein were exposed via a skin incision along the right groin. Blood pressure, electrocardiography, heart rate, and rectal temperature were monitored throughout the surgical procedure. Briefly, Blood pressure was measured with a pressure changer connected to a venous indwelling needle (24G) that was placed in the femoral artery. PowerLab 16/30 (AD-Instruments, Australia) was used to continuously monitor arterial blood pressure and the electrocardiogram. The 24G vein indwelling needle was placed in the right femoral vein for the continuous infusion of normal saline via a micro-perfusion pump at a velocity of 2 mL/h. After orotracheal intubation using a Teleflex 16G venous indwelling needle, a cervical median incision was made, and a firm ligature was placed on the main bronchi during mechanical ventilation in order to prevent air leakage.

After 10 min of mechanical ventilation, asphyxia was induced in the rats by stopping this mechanical ventilation. CA was defined as a drop in systolic blood pressure (SBP) to < 25 mmHg. Five minutes after CA (SBP < 25 mmHg), CPR was initiated. Adrenaline was injected (4 µg/100 g) through the right femoral venous indwelling needle together with external chest compression (200 bpm) and mechanical ventilation (tidal volume 0.65 mL/100 g, respiratory rate 80 bpm). ROSC was defined as a return of spontaneous sinus rhythm and the maintenance of a SBP > 60 mmHg for at least 10 min, as per the Utstein-style guidelines. If resuscitation failed, animals were excluded from the study. The orotracheal tube was removed after the return of spontaneous respiration. The catheters were removed and wounds stitched, with local infiltration using 1% lidocaine. At 24 h after ROSC, the rats were euthanized using an intraperitoneal injection of excess pentobarbital sodium(150 mg/kg). Subsequently, brain tissue was collected for further experiments.

### Preparation of BMSCs, lentiviral constructs, and transduction

#### Preparation of BMSCs

Isolation and cultivation of BMSCs were conducted as previously described with slight modifications [19]. Young SD rats weighing 100–120 g were euthanized, and their femurs and tibias were aseptically extracted in a laminar flow cabinet (Antai Airtech Co Suzhou, China). The bone cavities were thoroughly rinsed with Dulbecco’s Modified Eagle’s Medium/Ham’s F12 (DMEM/F12) without fetal bovine serum (FBS) (both sourced from Servicebio, Wuhan, China). This solution was collected in centrifuge tubes and centrifuged at 1000 rpm for 5 min. The supernatant was then removed. The pellet was resuspended in 6 mL of DMEM/F12 with 10% FBS, aliquoted into 25 cm^2^ tissue culture flasks, and cultured in a Tri-Gas incubator (Servicebio). Half of the medium was replaced after 24 h, the medium was fully changed after 48 h, and subsequent medium changes were performed every other day. The cells were detached using 2 mL of 0.25% pancreatin (Servicebio) containing 0.1 mM ethylenediaminetetraacetic acid (EDTA) for 2 min. Cell passaging (1:2) and subculturing were performed when cells were 90% confluent. P3 generation cells were utilized for further experimentation and transplantation. BMSCs were measured by flow cytometry (BD Bioscience) to analyze surface expression of CD29 (12594-1-AP, Proteintech), CD44 (GB113500, google), CD90 (30-H12, Proteintech), CD45 (60287-1-Ig, Proteintech), CD34 (SC-74499, Santa), CD11b (A1581, ABclonal). After the purity of BMSCs was confirmed to meet international standards through flow cytometry [20], the cells were adjusted to 1 × 10^6^ cells/mL for use in subsequent experiments.

#### Lentiviral constructs

The mRNA sequence of the rat *Rhot1* gene, identified as *Rattus norvegicus* ras homologous family member T1 (*Rhot1*) (NM_001107026.2), was obtained from NCBI. Primer sequences for the overexpression vector were designed following the Gibson reaction principle: forward 5′-TTGATAAGGTAACAAGCGATG-3′ and reverse 5′-TTTTGCTGAACACTCCACA-3′. Three pairs of shRNA sequences were designed using Vector Builder’s shRNA target design tool to target the *Rhot1* gene specifically: RHOT1 shRNA (1) - CGATGGATTCCTCTACTAAAT, RHOT1 shRNA (2) - CGATGAACTCAAAGATTAT, and RHOT1 shRNA (3) - GGAGACCATCCTTCCAATTAT. The synthesized sequences were provided to Yunzhou Biotechnology Co., Ltd. for vector construction and lentivirus packaging, generating the overexpression lentivirus pLV [Exp]-EGFP: T2A: Uro-EF1A > rRhot1[NM_001107026.2] (rRHOT1) and the control overexpression lentivirus pLV [Exp]-EGFP: T2A: Uro-EF1A > mCherry (vector1). Lentiviruses for silencing included pLV [shRNA]-EGFP: T2A: Puro-U6 > rRhot1 [shRNA#1] (sh1), pLV [shRNA]-EGFP: T2A: Puro-U6 > rRhot1[shRNA#2] (sh2), pLV [shRNA]-EGFP: T2A: Puro-U6 > rRhot1 [shRNA#3] (sh3), and the empty vector sh lentivirus pLV [shRNA]-EGFP: T2A: Puro-U6 > Scramble_shRNA (vector2). All designed lentiviruses included an EGFP fluorescent reporter gene and a puromycin resistance gene.

### RT-qPCR

Total RNA was extracted from the cells in each group using TRIzol (G3013, Servicebio), and the concentration of the total RNA was determined by measuring the OD260/OD280 ratio with an ultramicrospectrophotometer. Following the manufacturer’s instructions, the Revert Aid First Strand cDNA synthesis kit was utilized for cDNA synthesis. Each qPCR reaction mixture included a total volume of 20 µL, containing 5 µL of DNA template, 0.5 µL of forward primer (10 µmol/L), 0.5 µL of reverse primer (10 µmol/L), 10 µL of 2× SYBR Green qPCR SuperMix, and 4 µL of Nuclease-Free Water. The amplification reaction consisted of 95°C for 5 min followed by 40 cycles of denaturation at 95 °C for 30 s, annealing at 60 °C for 30 s, and extension at 72 °C for 30 s. The relative expression levels of genes in each group were calculated using 2^−ΔΔCt^ with *Gapdh* as the internal reference. The primer sequences for qRT-PCR were as follows: *Gapdh*—forward primer: 5′-GACAGCCGCATCTTCTTGT-3′, reverse primer: 5′-CTTGCGTGTGTAGATTCAT-3′; *Rhot1*—forward primer: 5′-CGCTCAAGCCTTCACTTGT-3′, reverse primer: 5′-GTGTTCACGTGGGTACAT-3′.

### CCK-8 assay

BMSCs in the logarithmic growth phase were divided into the BMSCs, BMSCs-miro^hi^, and BMSCs-miro^sh3^ groups. These cells were dissociated using trypsin and prepared as cell suspensions which were then plated in 96-well plates at 6000 cells/well, with 3 replicate wells per group (adding 150 µL of PBS to the outer wells to prevent evaporation). The cells were then incubated in a 37 °C in a 5% CO_2_ environment for 24, 48, or 72 h. At the appropriate time points, the original culture medium was aspirated from each well, and a 10% CCK-8-supplemented culture medium was added. Subsequently, 100 µL of the 10% CCK-8 culture medium was dispensed into each well and incubated for 2 h. Absorbance at 450 nm was measured using an ELISA reader. The proliferation rate was calculated as the percentage ratio of the absorbance of the experimental group to that of the control group, while the inhibition rate was computed as the percentage difference between 1 and the ratio of the absorbance of the experimental group to that of the control group.

### Brain tissue injury-related analyses

#### Flow cytometry analyses of apoptosis

The hippocampal tissue was digested with a trypsin cell digestion solution at 37 °C to prepare a single-cell suspension. Subsequently, the rat hippocampal neurons were washed three times with PBS, resuspended in PBS, and then stained following the protocol of the Annexin-V PI cell double-staining apoptosis detection kit (A211-02, Vazyme). Flow cytometry was utilized for the detection of cellular apoptosis.

#### Flow cytometry analyses of ROS

The hippocampal tissue was treated with a trypsin cell digestion solution at 37 °C to prepare a single-cell suspension. Following three PBS washes, rat hippocampal neurons were resuspended in PBS and stained as per the guidelines included in the reactive oxygen species detection kit (S0033S, Beyotime). Flow cytometry was employed to measure the cellular oxidative stress level.

### H&E staining

The brain tissue was fixed in 4% paraformaldehyde for 24 h, subsequently dehydrated, treated with a clearing agent, and then embedded in paraffin blocks. These blocks were later sectioned into thin slices. The sections underwent dewaxing, rehydration, and staining with hematoxylin and eosin (H&E), after which they were sealed. Observations of the staining were conducted under a microscope, and photographs of these sections were collected.

### Elisa

The hippocampal tissues were collected at 24 h post-ROSC to assess the levels of tumor necrosis factor α (TNF-α), interleukin 6 (IL-6), neuron-specific enolase (NSE), and IL-10. The tissues were rinsed with precooled PBS and subsequently homogenized. The homogenate was then centrifuged at 5000×*g* for 5 min, and the resulting supernatant was retained for further analyses. Detection of TNF-α, IL-6, and IL-10 levels was performed using Rat TNF-α, IL-6 and IL-10 ELISA kits (MM-0180R1, MM-0190R1, MM-0195R1; Meimian) in accordance with the manufacturer’s instructions. Optical density measurements were conducted using a microplate reader.

### Morris water maze

Spatial learning and memory were assessed using the Morris water maze (MWM) trial, which began 24 h post-ROSC. The MWM trial consisted of two parts: the hidden platform test (days 1 to 4) and the spatial exploration test (day 5). In the hidden platform test, a circular pool was divided into four quadrants, with a platform located 1 cm below the water surface in one quadrant. Rats were placed in the water from different starting points and tasked with finding the hidden platform. Each trial lasted 120 s, with rats placed on the platform for 15 s at the conclusion of the trial. Escape latency, the time taken by the rats to locate the platform and stay on it for 5 s, was recorded. If the rats failed to find the platform within 120 s, a maximum value of 120 s was assigned. On day 5, the spatial exploration test was conducted in the same pool but without the hidden platform. Frequency of crosses to the platform were recorded.

### Detection of mitochondrial transfer

#### Immunofluorescence analyses of BMSC mitochondria and hippocampal neuron co-localization

To label the mitochondria in BMSCs, the cells were incubated with MitoTracker Red CMXRos in a 37 °C incubator for 20 min. Subsequently, the cytoskeleton with these BMSCs was labeled with Phalloidin (C2201S, Beyotime), followed by three washes with PBS. The samples were then sealed using an anti-fluorescence quenching sealing solution containing DAPI (P0131-5 ml, Beyotime). Images were captured using a fluorescence microscope (BX53, Olympus).

The brains were fixed with 4% paraformaldehyde for 24 h. The fixed specimens underwent sequential sucrose gradient dehydration, OCT embedding, and were then sectioned at a thickness of 8 μm to prepare frozen sections. Following thawing, permeabilization, and sealing of these sections, they were incubated overnight with the primary anti-NeuN (AG5317, Beyotime) at 4 °C. The next day, the sections were washed with PBS and then incubated with an Alexa Fluor 488-conjugated secondary antibody (HZ0176, Huzhen) at room temperature for 1 h. After another round of washing with PBS, staining with DAPI-containing sealing agent (P0131, Beyotime) was conducted for 5 min, followed by sealing of these sections. The sections were observed under a fluorescence microscope, and images were captured.

#### Immunofluorescence detection of TOMM20 expression in hippocampal neurons

Sections were incubated with the primary anti-Tomm20 (AF5206, Affinity), followed by the 488-conjugated secondary antibody (B100805, Baiqiandu) and the CY3-conjugated secondary antibody (B100802, Baiqiandu). Subsequently, the slices were sealed with a DAPI-containing sealing agent, observed, and photographed under a fluorescence microscope.

### Mitophagy-related analyses

#### Electron microscopy

The fixed hippocampal tissues were washed with PBS for 45 min, followed by fixation with 1% osmium tetroxide for 2 h. Subsequently, the tissue was subjected to gradient ethanol dehydration, epoxy resin infiltration, embedding, and ultra-thin sectioning (60–80 nm) with uranium and lead staining to visualize the cellular structures. The number of autophagosomes inside the cells was then photographed and recorded under a transmission electron microscope (HT7800, Hitachi).

### Western blotting

To assess protein expression, rat hippocampal tissues were lysed in RIPA buffer (BL504A, Biosharp) containing 1% PMSF (G2008-1ML, Servicebio) on ice. Total protein concentrations for each group were measured using the BCA (G2026-1000T, Servicebio) method. Samples were boiled in a water bath for 10 min, followed by separation via 12% SDS-PAGE (P0012A, Beyotime) and transfer onto a polyvinylidene fluoride membrane (FFP70, Beyotime). Blocking was performed with 5% skim milk at room temperature for 2 h. Blots were then incubated for 12 h at 4 °C with gentle shaking with the following antibodies: anti-LC3 (14600-1-AP, proteintech), anti-P62 (ab56416, abcam), anti-PINK1 (A11435, ABclonal), anti-parkin (A11172, ABclonal), anti-Tomm20 (ab186735, abcam), anti-ATG5 (39202, SAB), anti-Miro1 (A22469, ABclonal), and anti-β-actin (BS-0061R, BIOSS). Samples were then incubated with a horseradish peroxidase-labeled secondary antibody (5220-0341, Seracare) at room temperature for 2 h, and developed using an electrochemiluminescence (MA0186, meilunbio) method.

### Mitochondrial quality-related analyses

#### Measurement of mitochondrial membrane potential

The hippocampal tissue was digested with a trypsin cell digestion solution at 37 °C to prepare a single-cell suspension. Subsequently, the rat hippocampal neurons were washed three times with PBS, followed by resuspension in PBS and staining based on the protocol provided with the JC-1 kit (C2006, Beyotime). Analyses of the mitochondrial membrane potential of these cells were conducted using flow cytometry.

#### Flow cytometry analyses of MtROS levels

Hippocampal tissue was digested with a trypsin cell digestion solution at 37 °C to prepare a single-cell suspension, which was subsequently rinsed three times with PBS. A suitable amount of diluted Mito sox Red (M36008, Invitrogen) was then added. After a 20-min incubation at 37 °C, the sample was centrifuged and washed three times with serum-free medium to eliminate any unabsorbed Mito sox Red. Fluorescence intensity was assessed using a flow cytometer.

### Determination of ATP content

ATP levels in each group were assessed as per the protocol and calculation formula provided with the ATP content test kit (A095-1-1, Jiancheng).

### Neurological functional scores

Neurological function was evaluated as per the established literature [21], employing the Neurological Deficit Score (NDS) to appraise the condition of post-ROSC rats at 6, 12, 24, 72, and 168 h. Two proficient evaluators, blinded to the group assignments for these rats, independently performed the evaluations, and the mean scores from their assessments were recorded. Evaluation criteria comprised parameters such as consciousness, basic reflexes, motor function, and sensory perception, among others. In total, 7 scoring components were considered, for a maximum possible score of 80 points. A score of 80 was indicative of normal health, while a score of 0 signified death.

### Statistical analyses

Statistical analyses were performed using GraphPad Prism 9.0, and the results are displayed as means ± standard deviation (SD). Each dataset included a minimum of three biological replicates. The NDS scores were assessed using repeated measures analyses of variance (ANOVAs) while the other data were compared through one-way ANOVAs followed by Tukey’s HSD (honestly significant difference) test. A significance level of *P* < 0.05 was employed to determine statistical significance.

## Results

### Rat physiological parameters during cardiopulmonary resuscitation

There were no notable variations in the baseline data among the rats in different experimental groups before the induction of asphyxia (*P* > 0.05). Additionally, there were no statistically significant differences in the duration of asphyxia, time to spontaneous restoration of cardiac rhythm, and adrenaline dosage when comparing the CPR-PBS group with the CPR-BMSCs group (*P* > 0.05). Similarly, prior to the initiation of asphyxia, there were no apparent variations in the baseline data, duration of asphyxia, time to spontaneous cardiac rhythm recovery, or adrenaline dosages among the BMSCs, BMSCs-miro^hi^, and BMSCs miro^lo^ groups (*P* > 0.05) (Table 1).

**Table 1:** Physiological parameters of rats during cardiopulmonary resuscitation

Group	Body weight (g)	Heart rate, (bpm)	Mean atrial pressure, (mmHg)	Time before cardiac arrest, (s)	ROSC time, (s)	Heart rate after ROSC, (bpm)	Mean atrial pressure after ROSC, (mmHg)	Adrenaline dose, (µg)
Sham	307 ± 15.8	355 ± 13.3	96.8 ± 5.7	–	–	–	–	
CA/CPR	310 ± 15.7	352 ± 17.2	94.3 ± 4.05	213.1 ± 17.8	98.8 ± 13.1	70.9 ± 8.4	255 ± 18.2	12.45 ± 0.598
BMSCs	308 ± 15.1	359 ± 16.4	97.9 ± 6.08	216.5 ± 24.9	90.3 ± 7.6	72 ± 5.2	263 ± 16.3	12.3 ± 0.632
BMSCS-miro1^hi^	313 ± 16.8	353 ± 14.5	95.5 ± 5.10	211.6 ± 20.1	89.3 ± 14.4	73 ± 4.9	257 ± 12.8	12.55 ± 0.598
BMSCS-miro1^l^°	305 ± 15.7	364 ± 20.3	94.7 ± 5.85	218.1 ± 12.7	93.0 ± 11.6	75 ± 2.4	254 ± 15.5	12.25 ± 0.634
F值	0.347	0.909	0.763	0.238	1.408	1.082	0.691	0.499
*P*值	0.845	0.467	0.555	0.869	0.256	0.369	0.564	0.685

### Lentivirus-mediated preparation of rat BMSCs with Miro1 overexpression or Silencing

**Fig. 2:**
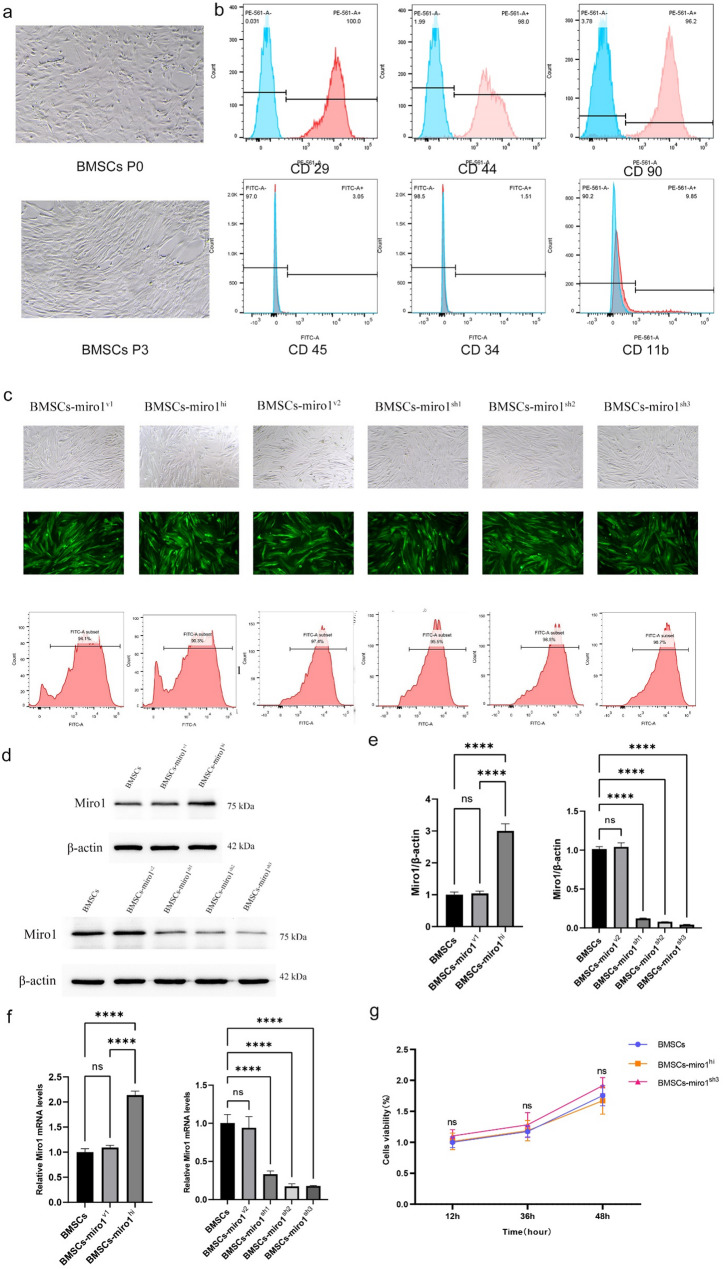
Characteristics of cultured rat BMSCs and confirmation of lentivirus-mediated overexpression and knockdown of Miro1. **a** Representative images of cells from passages 0 (P0) and 3 (P3). **b** Flow cytometry results indicating that BMSCs from P3 were positive for CD29, CD44, and CD90, and were negative for CD11b, CD34, and CD45. () Fluorescence images showing that transduced BMSCs co-express Green Fluorescent Protein (EGFP) with corresponding quantification of transduction rates in the BMSCs, BMSCs-miro^v1^, BMSCs-miro^hi^, BMSCs-miro^v2^, BMSCs-miro^sh1^, BMSCs-miro^sh2^, and BMSCs-miro^sh3^ groups. **d**,** e** Western blotting results showing the relative abundance of Miro1 in the BMSCs, BMSCs-miro^v1^, BMSCs-miro^hi^, BMSCs-miro^v2^, BMSCs-miro^sh1^, BMSCs-miro^sh2^, and BMSCs-miro^sh3^ groups. **f** RT-qPCR results showing the relative expression of *Miro1* in the BMSCs, BMSCs-miro^v1^, BMSCs-miro^hi^, BMSCs-miro^v2^, BMSCs-miro^sh1^, BMSCs-miro^sh2^, and BMSCs-miro^sh3^ groups. **g** Cell Counting Kit-8 (CCK-8) analysis results showing the viability of cells in the BMSCs, BMSCS-miro^hi^, and BMSCS-miro^sh3^ groups (*n* = 3). All data are presented as the means ± SD. *****P < 0.0001. ns *P* > 0.05

After isolating P3 rat BMSCs in vitro, their morphology was examined using an inverted microscope. This observation revealed that the adherent cells displayed uniform star-shaped or polygonal morphology and exhibited rapid growth, signifying successful cell purification for subsequent experiments (Fig. 2a). Flow cytometry was used to detect surface markers on these cultured P3 rat BMSCs as an indicator of cell purity. This analysis demonstrated strong positive expression levels of CD29 (100%), CD44 (98%), and CD90 (96.2%), while CD45 (3.05%), CD34 (1.51%), and CD11b (9.85%) were found to be consistently negatively expressed (Fig. 2b). In addition, in vitro differentiation of BMSCs were conducted:Osteogenic differentiation (supplement material 1).

The efficiency of lentiviral transfection in BMSCs was evaluated using fluorescence microscopy, flow cytometry, western blotting, and RT-qPCR to detect Green Fluorescent Protein (EGFP), Miro1, and mRNA expression. Following lentiviral infection at a multiplicity of infection (MOI) of 100, EGFP fluorescence indicative of transfection was observed in all BMSC groups (BMSCs-miro^v1^, BMSCs-miro^hi^, BMSCs-miro^v2^, BMSCS-miro^sh1^, BMSCs-miro^sh2^, and BMSCs-miro^sh3^) under a fluorescent inverted microscope, with positive transduction rates ranging from 90.3 to 98.7% (Fig. 2c). The western blotting analysis demonstrated the protein-level expression of Miro1 across all cell groups, Significantly increased Miro1 protein levels were observed in the BMSCs-miro^hi^ group compared to BMSCs, while no substantial differences were noted between BMSCs and BMSCs-miro^v1^. Conversely, reduced Miro1 protein expression was observed in the BMSCs-miro^sh1^, BMSCs-miro^sh2^, and BMSCs-miro^sh3^ groups, with BMSCs-miro^sh3^ exhibiting the most pronounced decrease (Fig. 2d, e). The Real-time PCR results aligned with the western blotting results, confirming the efficient lentiviral transfection of BMSCs (Fig. 2f). Cell proliferation, assessed via the CCK-8 method, revealed no significant differences among BMSCs, BMSCs-miro^hi^, and BMSCs-miro^sh3^ (Fig. 2g), indicating stable proliferation following lentiviral transfection. The BMSCs-miro^sh3^ group was redesignated as BMSCs-miro^lo^, demonstrating the successful generation of normal BMSCs, Miro1-overexpressing BMSCs (BMSCs-miro^hi^), and Miro1-knockdown BMSCs (BMSCs-miro^lo^).

### BMSC transplantation restores mitochondrial quality through A dual mechanism involving mitochondrial transfer and mitophagy

#### BMSC transplantation provides healthy mitochondria to hippocampal neurons of CPR model rats

**Fig. 3:**
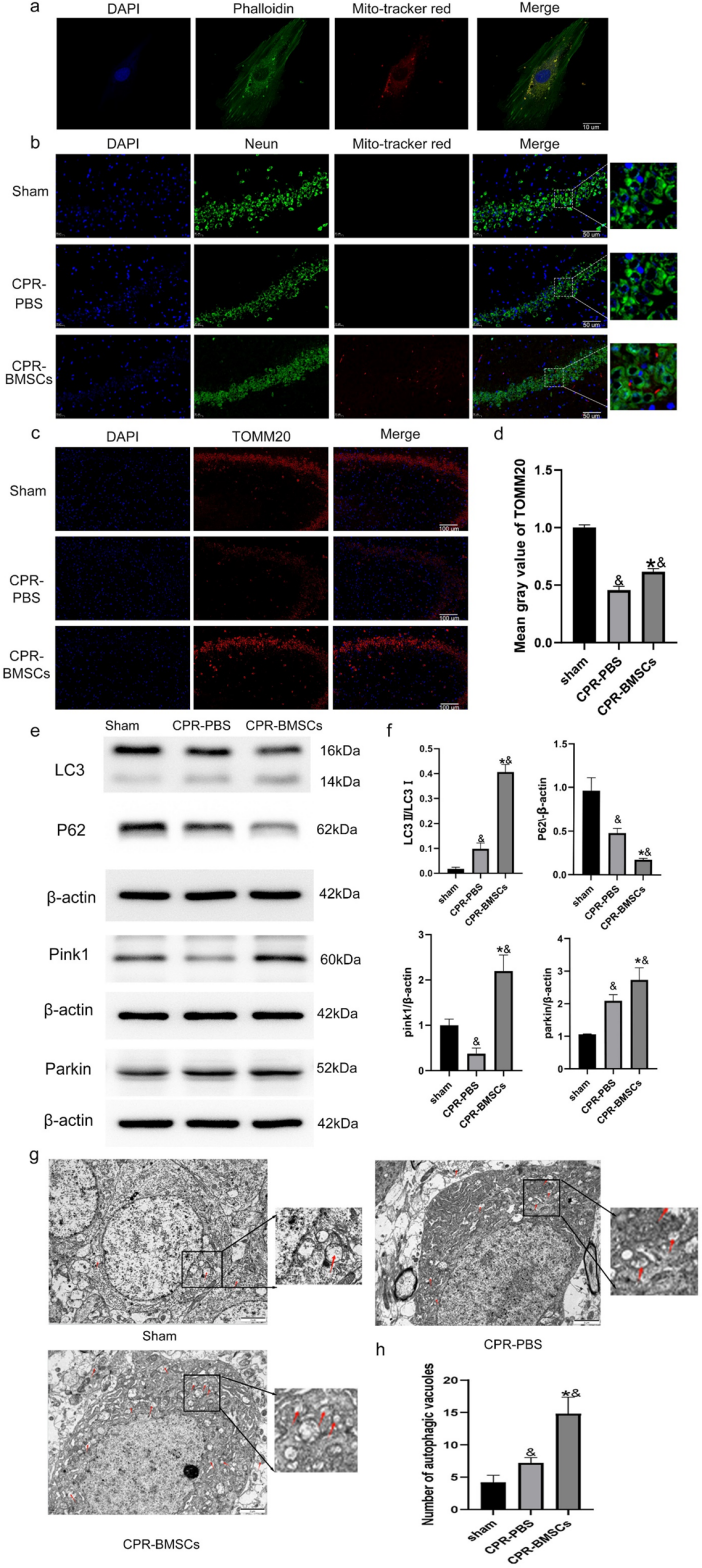
BMSC transplantation can provide healthy mitochondria and mediate Pink1 Parkin mitophagy pathway to hippocampal neurons. **a** Mitochondrial BMSCs were pre-labeled with MitoTracker. **b** Immunofluorescence analyses showing the presence of BMSC-derived mitochondrial pre-labeled with MitoTracker in vitro in hippocampal neurons of rats after CPR. **c**,** d** Immunofluorescence analyses of TOMM20 in hippocampal neurons of rats after CPR (*n* = 3). **e**, **f.** Western blotting results showing expression of LC3, P62, Pink1, and Parkin in post-resuscitation rats (*n* = 3). **g**, ** h** Ultrastructural images of autophagic vacuoles (red arrows) in rat hippocampal neurons (*n* = 5). All data are presented as means ± SD. *P < 0.05 vs. the CPR-PBS group; &*P* < 0.05 vs. the Sham group

The successful transplantation of BMSCs to deliver healthy mitochondria to hippocampal neurons of rats after CPR was achieved. Briefly, mitochondria within the isolated BMSCs were labeled using MitoTracker Red CMXRos, with the cytoskeleton having additionally been labeled using Phalloidin to evaluate MitoTracker Red CMXRos labeling effectiveness. Fluorescence microscopy visualization confirmed that labeling was successful, (Fig. 3a). Hippocampal neurons were marked with NeuN to assess the capability of exogenous BMSCs to traverse the blood-brain barrier and provide healthy mitochondria to damaged hippocampal neurons. Immunofluorescence analyses revealed that 24 h post-ROSC, mitochondria originating from exogenous BMSCs were internalized by and incorporated into hippocampal neurons within the hippocampus (Fig. 3b). Further immunofluorescence analyses using TOMM20 were conducted across the sham, CPR-PBS, and CPR-BMSCs groups, demonstrating a notable rise in the overall mitochondrial content in hippocampus of the CPR-BMSCs group compared to the CPR-PBS group within 24 h after ROSC (Fig. 3c, d). In addition, the relative mtDNA content between the Sham group and the CPR-BMSCs group were determined (supplement material 1). Together, these findings suggest that the administration of exogenous BMSCs via the femoral vein effectively transports healthy mitochondria to hippocampal neurons in CPR model rats, enriching the mitochondrial population within the hippocampus.

#### BMSCs mediate the activation of the Pink1 parkin mitophagy pathway

Western blotting analyses of hippocampal neurons revealed that at 24 h after ROSC, the expression of LC3-II/ LC3-I, PINK1, and Parkin, which are positively correlated with mitophagy, was significantly increased in the CPR-BMSCs group compared to the CPR-PBS group (*P* < 0.05), while the expression of p62 (a protein negatively correlated with mitophagy) was significantly decreased (*P* < 0.05) (Fig. 3e, f). Transmission electron microscopy analyses of the number of autophagosomes in hippocampal neurons yielded consistent results. Specifically, relatively few autophagosomes with a bilayer membrane structure were observed in the hippocampal neurons of the Sham group, while significantly more were evident in the CPR-PBS group relative to the Sham group (*P* < 0.05), and significantly more autophagosomes were observed in the CPR-BMSCs group relative to the Sham and CPR-PBS groups (*P* < 0.05). Based on these results, exogenous BMSCs were transplanted via femoral vein injection to enhance mitophagy in rat hippocampal neurons after CPR (Fig. 3g).

#### Transplantation of BMSCs improves mitochondrial mass of hippocampal neurons in rats after CPR

**Fig. 4:**
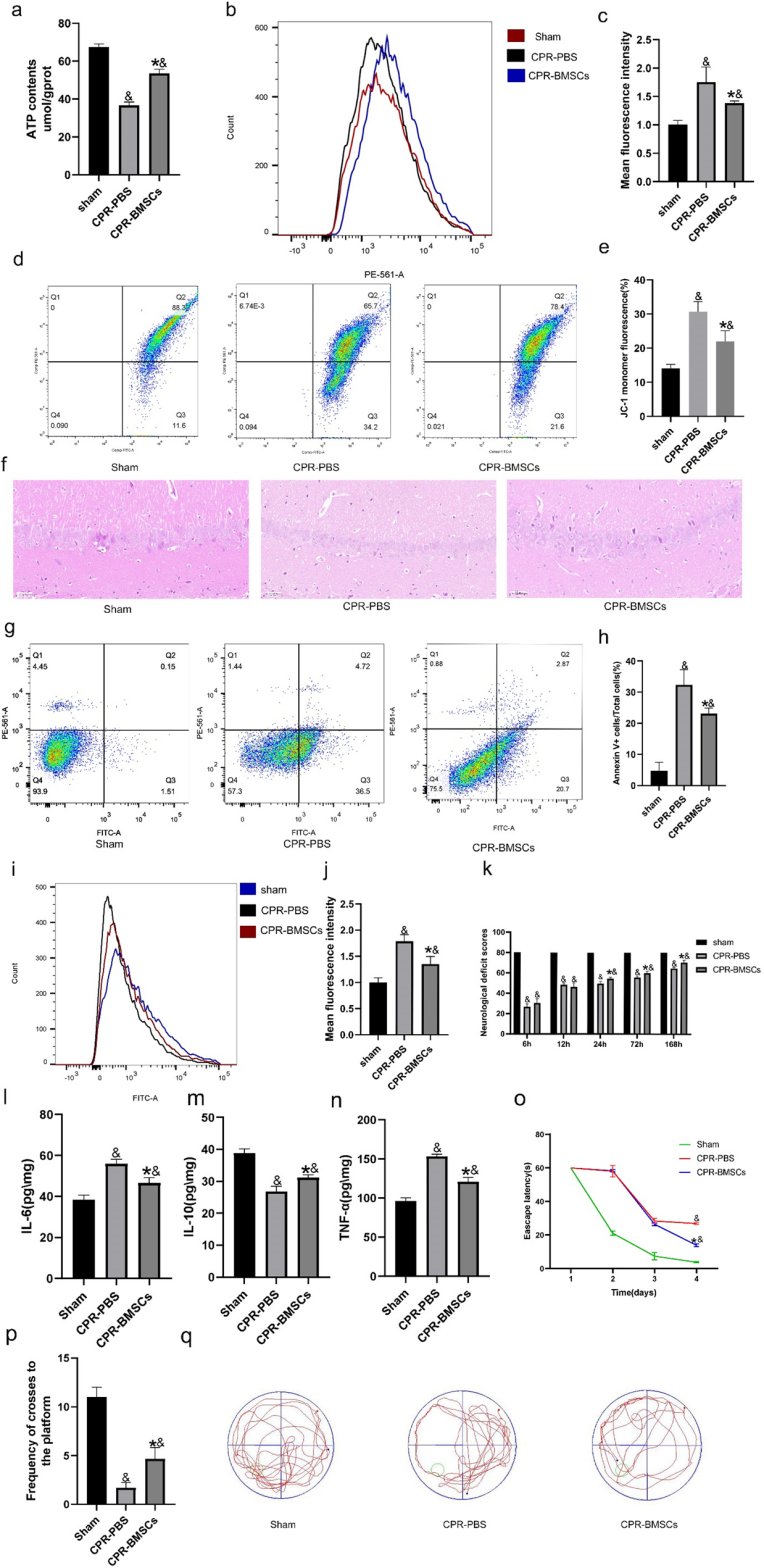
BMSC transplantation can improve mitochondrial quality and alleviate hippocampal neuron injury after CPR. **a** ATP levels were quantified in hippocampal neurons (*n* = 5). **b**, **c** mtROS levels were measured by flow cytometry in hippocampal neurons (*n* = 5). **d**, **e** Mitochondrial membrane potential was detected using JC-1 staining (*n* = 5). **f** BMSCs ameliorated pathological injury in the cerebral hippocampus in rats following global cerebral I/R. Scale bar = 50 μm (*n* = 5) **g**, ** h** Hippocampal neuron apoptosis was detected via Annexin-V‐FITC/PI staining, and cellular apoptosis was detected by flow cytometry. (*n* = 5–6). **i**, **j**BMSCs reduced ROS levels in hippocampal neurons after cardiopulmonary resuscitation as measured by flow cytometry (*n* = 5). Data are means ± SD. **k** Neurological deficit scores showing that BMSCs improved the recovery of neurologic function after resuscitation (*n* = 10). **l**–**n** Levels of, IL-6, IL-10, and TNF-α in hippocampal tissue. (*n* = 3) **o** Escape latency during the hidden platform test from day 1 to day 4. **p**, **q** Frequency of crosses to the platform and representative track plots of each group during the space exploration test. All data are presented as means ± SD. *P < 0.05 vs. the CPR-PBS group; &*P* < 0.05 vs. the Sham group

The above results suggest that transplantation of BMSCs following CPR in rats plays a significant role in improving mitochondrial function in hippocampal neurons. Moreover, compared to the Sham group, the CPR-PBS group exhibited significantly reduced ATP content in hippocampal neurons, while the CPR-BMSCs group also exhibited reduced ATP content, albeit with a significant increase in ATP content in neuronal cells in the CPR-BMSCs group compared to the CPR-PBS group. (Fig. 4a). Mitochondrial membrane potential and mitochondrial oxidative stress levels were next examined in these assays, revealing that both were increased in the CPR-PBS group relative to the Sham group, whereas the transplantation of BMSCs led to a reduction in both mitochondrial membrane potential and oxidative stress levels in hippocampal neurons (Fig. 4b, c, d, e). Overall, these findings demonstrate that injecting exogenous BMSCs via the femoral vein can help alleviate mitochondrial damage resulting from global cerebral ischemia after CPR and contribute to the restoration of mitochondrial quality in hippocampal neurons.

## Transplanting BMSCs alleviates hippocampal neuronal damage in rats after CPR

Histological analyses using H&E staining were next performed to examine the pathological conditions of the hippocampus. (Fig. 4f). These results revealed that rats in the Sham group exhibited an orderly arrangement of neurons with shallow cytoplasmic staining, round nuclei, and no significant pathological damage. In contrast, the CPR-PBS group showed disordered neuron arrangement, reduced cell numbers, and deep staining of the cell nuclei, consistent with significant pathological damage. However, the CPR-BMSCs group demonstrated increased hippocampal neuron density, minimal disorder in terms of cell arrangement, and only a small number of apoptotic damaged cells. Flow cytometry was further used to evaluate hippocampal neuronal apoptosis and levels of ROS to assess brain tissue damage (Fig. 4g–j). The CPR-BMSCs group exhibited a significant decrease in neuronal apoptosis rate and ROS levels compared to the CPR-PBS group. Additionally, neurological function was assessed at 6, 12, 24, 72, and 168 h post-ROSC to reflect neurological deficits (Fig. 4k). No significant differences in NDS were observed among the three groups at 6 and 12 h post-ROSC. However, at 24, 72, and 168 h post-ROSC, the NDS scores of rats in the CPR-BMSCs group were higher than those of rats in the CPR-PBS group. We found that proinflammatory cytokines TNF-α and IL-6 were decreased (Fig. 4l, n), while IL-10 were increased (Fig. 4m) significantly in the CPR-BMSCs group. In the MWM assessment, the CPR-BMSCs group exhibited a faster reduction in escape latency in the hidden platform test (Fig. 4o) and frequency of crosses to the platform during the spatial exploration test (Fig. 4p, q) in comparison to the CPR-PBS group. The above results indicate that the transplantation of exogenous BMSCs through the femoral vein could mitigate damage to rat neurons following CPR.

### Miro1 optimizes the BMSC-mediated restoration of mitochondrial homeostasis

#### Miro1 enhances the efficiency of mitochondrial transfer from BMSCs

**Fig. 5:**
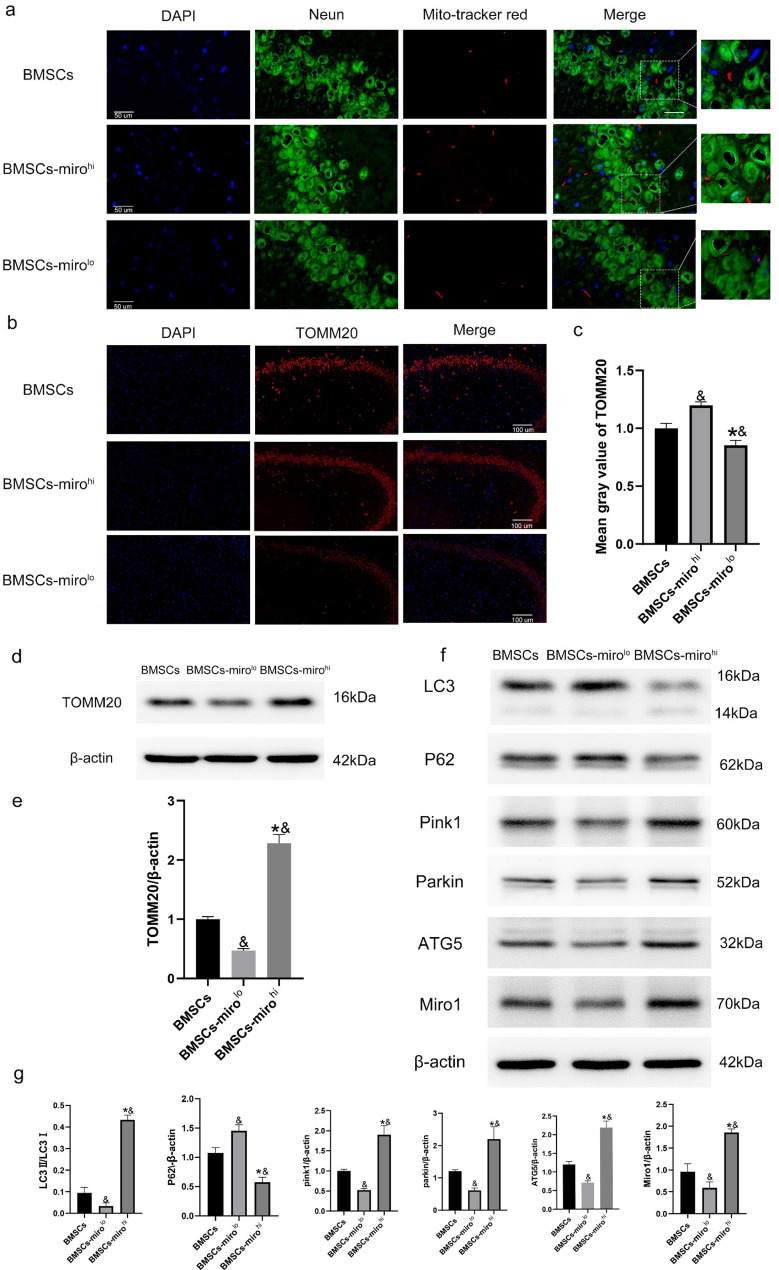
Miro1 enhances BMSC-mediated mitochondrial transfer and mitophagy. **a** Immunofluorescence results showing the presence of mitochondria from BMSCs-miro^hi^ cells that had been pre-labeled with MitoTracker in vitro in hippocampal neurons of rats after CPR. **b**, **c** Immunofluorescence staining of TOMM20 in hippocampal neurons of rats after CPR (*n* = 3). **d**, **e** Western blotting results showing the expression of TOMM20 in post-resuscitation rats (*n* = 3). *P < 0.05 vs. the BMSCs-miro^hi^ group. &*P* < 0.05 vs. the BMSCs group. **f**, **g** Western blotting results showing the expression of LC3, P62, Pink1, Parkin, ATG5, and Miro1 in post-resuscitation rats (*n* = 3). All data are presented as the means ± SD. *P < 0.05 vs. the BMSCs-miro^lo^ group. &*P* < 0.05 vs. the BMSCs group

Immunofluorescence analyses were conducted for samples from rats in the BMSCs, BMSCs-miro^hi^, and BMSCs-miro^lo^ groups as the Sham, CPR-PBS, and CPR-BMSCs group. At 24 h after ROSC, the BMSCs-miro^hi^ group exhibited a considerable increase in the internalization of mitochondria within the hippocampus compared to the other BMSCs groups. Conversely, the number of internalized mitochondria in the BMSCs-miro^lo^ group, in which Miro1 had been knocked down in BMSCs, was significantly reduced (Fig. 5a). These findings were corroborated by the results obtained from immunoblotting and immunofluorescence analyses of TOMM20 content in the hippocampus. Notably, at 24 h post-ROSC, a marked increase in the total number of healthy mitochondria within the hippocampus was observed in the BMSCs-miro^hi^ group in contrast to the BMSCs group, with a significant decrease in the total healthy mitochondria count in the hippocampus of the BMSCs-miro^lo^ group (*P* < 0.05) (Fig. 5b, c, d, e). These results suggest a potential role for Miro1 as a driver of more efficient mitochondrial transfer by BMSCs.

#### Miro1 enhances BMSCs-mediated activation of the Pink1-parkin mitophagy pathway

Western blotting analyses of hippocampal neurons revealed that at 24 h after ROSC, the expression of LC3-II, PINK1, Parkin, and ATG5 was significantly increased in the BMSCs-miro^hi^ group compared to the BMSCs group (*P* < 0.05), while the expression of p62 was significantly decreased compared to the BMSCs group (*P* < 0.05) (Fig. 5f, g). Miro1 thus enhances BMSC-mediated activation of the Pink1-Parkin mitophagy pathway.

#### Miro1 facilitates further improvements in mitochondrial quality

**Fig. 6:**
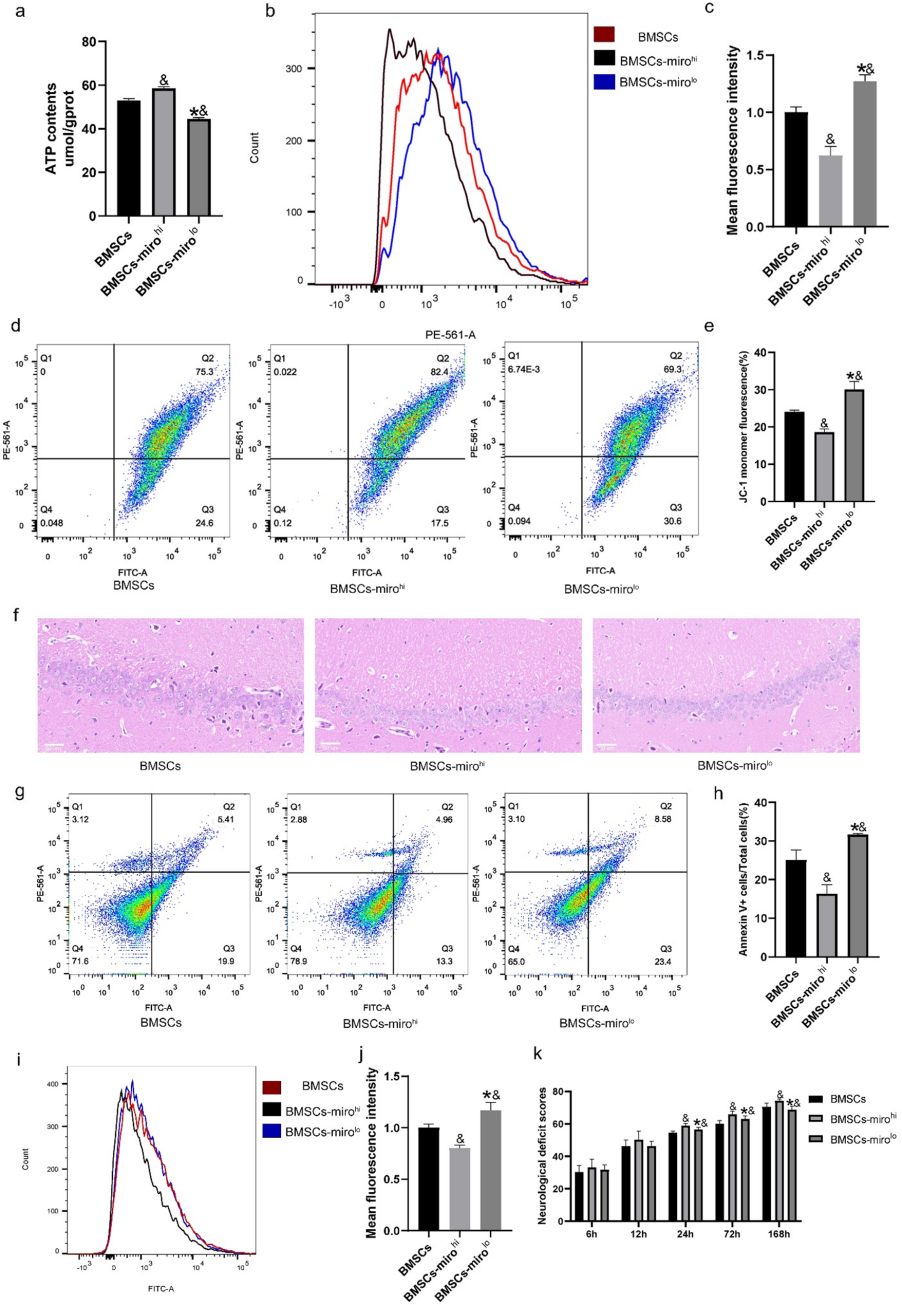
Miro1 enhances the therapeutic effects of BMSCs and reduces brain injury. **a** ATP levels were quantified in hippocampal neurons (*n* = 3) **b**, **c** mtROS levels were determined by flow cytometry in hippocampal neurons. (*n* = 3). **d**, **e** Mitochondrial membrane potential was detected via JC-1 staining (*n* = 3). **f** BMSCs-miro^hi^ treatment ameliorated pathological injury in the cerebral hippocampus in rats with global cerebral I/R. Scale bar = 50 μm (*n* = 5). **g**, **h** BMSCs-miro^hi^ were able to reduce neuronal cell apoptosis in rats after CPR as measured via flow cytometry (*n* = 5). **i**, **j** BMSCs-miro^hi^ treatment reduced ROS levels in hippocampal neurons after CPR as measured via flow cytometry (*n* = 5). **k** Neurological deficit scores showing that BMSCs-miro^hi^ improved the recovery of neurologic function after resuscitation (*n* = 10). All data are presented as the means ± SD. *P < 0.05 vs. the BMSCs-miro^hi^ group. &*P* < 0.05 vs. the BMSCs group

Analyses of ATP levels in the hippocampal neurons from these three groups revealed a significant increase in ATP content in hippocampal neurons compared to the BMSCs group (*P* < 0.05), whereas the BMSCs-miro^lo^ group exhibited a marked decrease in ATP content in neurons (*P* < 0.05) (Fig. 6a). Moreover, the BMSCs-miro^hi^ group exhibited substantially reduced mtROS levels in hippocampal neurons relative to the BMSCs group (*P* < 0.05), while the BMSCs-miro^lo^ group exhibited a slight increase in mtROS content and mitochondrial membrane potential levels (*P* < 0.05) (Fig. 6b–e). These results suggest that Miro1 contributes to the enhancement of recovery from mitochondrial damage following global cerebral ischemia-reperfusion after CPR.

#### Miro1 enhances the ability of BMSCs to protect against brain injury

H&E staining was utilized to assess the pathological conditions of the hippocampus in experimental rats (Fig. 6f). Despite exhibiting signs of CA/CPR damage, the BMSCs-miro^hi^ group displayed a relatively more organized hippocampal cell morphology compared to the BMSCs group. In contrast, the therapeutic impact of BMSC administration in the BMSCs-miro^lo^ group was not as pronounced. BMSCs-miro^hi^ treatment led to decreased production of abnormal hippocampal neurons, resulting in reduced cell damage at 24 h post-injection. Flow cytometry was employed to analyze hippocampal neuronal apoptosis and ROS levels, reflecting the extent of brain tissue damage (Fig. 6g–j). In contrast with the BMSCs group, the BMSCs-miro^hi^ group exhibited a pronounced reduction in neuronal apoptosis rates and ROS levels (*P* < 0.05), whereas the BMSCs-miro^lo^ group exhibited an increase in both parameters (*P* < 0.05). Furthermore, analyses of NDS scores at 24 h post-transplantation indicated that the BMSCs-miro^hi^ group achieved superior neural function scores as compared to the BMSCs group (*P* < 0.05). Similarly, the BMSCs group exhibited significantly enhanced neurological function compared to the BMSCs-miro^lo^ group (Fig. 6k). The findings suggest that BMSCs-miro^hi^ transplantation exerts a protective effect against cerebral ischemia-reperfusion injury following resuscitation that surpasses the neuroprotective benefits of unmodified BMSC transplantation.

## Discussion

In this study, we investigated the feasibility of BMSC transplantation via femoral vein injection as a potential treatment for I/R injury following CA. Our investigation revealed several key findings. First, CPR-induced global brain I/R injury in rats resulted in a significant increase in neuronal cell apoptosis, the disruption of mitochondrial homeostasis, and the impairment of neurological function. Second, BMSCs demonstrated a dual protective mechanism by facilitating the transfer of healthy mitochondria from exogenous sources and enhancing the autophagy of damaged mitochondria post-CPR, leading to improved mitochondrial function in hippocampal neurons and reduced neuronal apoptosis. Third, the overexpression of Miro1 in BMSCs was found to enhance mitochondrial transfer efficacy and promote mitophagy in BMSCs, thereby enhancing the therapeutic potential of BMSCs.

In 2008, researchers first conducted the right atrial injection of BMSCs to investigate their neuroprotective impact on CPR in rats [22]. This study revealed that the transplanted BMSCs penetrated the blood-brain barrier, dispersed across various brain regions, mitigated brain damage resulting from CPR, and restored neurological function. However, the precise underlying mechanisms remain elusive. Although the direct lateral ventricular administration of BMSCs offers superior efficacy as a means of delivering cells to the injured brain and maximizing therapeutic benefits [23], the invasiveness of this procedure poses risks of increased rat mortality and may impede future clinical applications. A significant proportion of intravenously injected BMSCs can effectively migrate to the damaged brain areas following CPR, particularly in the hippocampus and cortex [11]. Consequently, the choice was made to continue BMSC transplantation via the femoral vein in the present study.

Mitochondrial transfer involves the translocation of fully functional mitochondria through diverse pathways to the site of ischemic injury. This process replaces damaged mitochondria and mtDNA [24], amplifying neuronal activity and self-healing capacity, thus averting the cascading effects of mitochondrial damage in situ and mitigating I/R injury [25]. Mitochondrial transfer is a pivotal mechanism through which BMSCs can help repair tissue cell damage [26]. Both in vivo [27] and in vitro experiments [28] have confirmed that MSCs can transfer their mitochondria to impaired cells, exerting protective effects through methods such as intravenous injection or co-culture. High levels of expression of Miro1 enhance mitochondrial motility, effectively boosting the efficiency of healthy mitochondria transfer to damaged cells, ultimately reducing neuronal apoptosis and playing a protective role in neurological disorders like traumatic brain injury [29] and Parkinson’s disease [30]. The immunofluorescence analyses performed herein confirmed that following the intravenous administration of BMSCs, some mitochondria from these BMSCs were internalized by hippocampal neurons, resulting in the augmented presence of healthy mitochondria in hippocampal cells after CPR. Moreover, Miro1, as a key protein essential for mitochondrial transfer, localizes on the outer membrane of eukaryotic mitochondria. Miro1 plays a vital role in bolstering the efficacy of BMSCs-mediated mitochondrial transfer, facilitating the translocation of healthy mitochondria from BMSCs to impaired neuronal cells. It is hypothesized that the I/R injury that occurs during CA and CPR disrupts the blood-brain barrier, potentially allowing exogenous BMSCs to traverse the barrier and gain entry into the hippocampus, thereby delivering beneficial mitochondria to dysfunctional hippocampal neurons. In essence, the transplanted BMSCs contribute healthy mitochondria to hippocampal neurons through mitochondrial transfer, with Miro1 substantially enhancing the transfer process efficiency, consequently elevating the healthy mitochondrial count in the hippocampus.

The process of mitophagy involves the selective recognition and clearance of damaged mitochondria through autophagy, serving as a crucial mechanism for maintaining mitochondrial quality and normal cellular physiological function [31]. Moderate mitophagy acts as a neuroprotective mechanism by efficiently clearing damaged mitochondria, mitigating ROS production through negative feedback, and attenuating oxidative stress-associated damage [32]. Normally, PINK1 (PTEN-induced kinase 1) is transported to the inner mitochondrial membrane (IMM) and undergoes hydrolysis by specific proteases in a membrane potential-dependent manner [33]. PINK1 is then degraded through the ubiquitin protease system and maintained at a low level. Changes in mitochondrial membrane potential impede the effective entry of PINK1 into the IMM, leading to its accumulation on the outer mitochondrial membrane (OMM) [34]. PINK1 accumulation on the OMM leads to its autophosphorylation, activating PINK1, which in turn phosphorylates and activates Parkin. This activation recruits Parkin to induce mitochondrial depolarization. Subsequently, Parkin ubiquitinates the mitochondrial protein Miro1, segregating damaged mitochondria and facilitating their entry into the proteasome for degradation [35]. After transplanting BMSCs through the femoral vein, our study demonstrated an increase in CA-induced PINK1/Parkin expression and a significant rise in the number of autophagosomes observed under electron microscopy, indicating heightened mitophagy. Additionally, the PINK1/Parkin expression in BMSCs transfected with Miro1 was further augmented post-transplantation. These findings suggest that BMSC transplantation may facilitate the removal of damaged mitochondria in hippocampal cells through PINK1/Parkin-mediated mitophagy, thereby exerting a neuroprotective effect. Furthermore, Miro1 can enhance the efficacy of BMSC-mediated PINK1/Parkin mitophagy, potentially boosting its therapeutic impact.

Neuronal apoptosis is a key mechanism underlying neuronal damage and compromised neural function following cerebral ischemia and hypoxia [35]. During ischemia-reperfusion injury, mitochondria serve as the primary source of uncontrolled ROS production, leading to alterations in mitochondrial membrane potential via the disruption of the mitochondrial permeability transition pore [36]. Mitochondrial dysfunction, in turn, is a primary consequence of unrestrained ROS [37, 38]. These interconnected characteristics create a detrimental cycle in which oxidative stress-induced damage triggers heightened ROS production within mitochondria, leading to further mitochondrial damage and degradation of the intracellular environment [39]. Cerebral blood flow interruption-induced ischemia and reperfusion can induce cellular damage responses such as energy disturbances, acidosis, enhanced release of excitatory amino acids, generation of free radicals, and upregulation of apoptotic gene expression, culminating in apoptosis and necrosis [40]. Thus, preserving a robust mitochondrial functional network is imperative for responding to physiological adaptation and oxidative stress. In this study, the transplantation of BMSCs via femoral vein injection was shown to restore mitochondrial membrane potential levels, decrease mitochondrial oxidative stress content, increase ATP levels, and enhance mitochondrial quality in hippocampal neurons. These effects ultimately lead to a reduction in the apoptosis rate of hippocampal neurons. Moreover, the presence of Miro1 was found to augment the therapeutic impact of BMSCs in this experimental context, thereby mitigating brain injury post-CPR. These findings suggest that the transplantation of BMSCs could potentially alleviate tissue damage in the central nervous system by ameliorating oxidative stress-related damage and restoring mitochondrial quality following CA.

Limitations of this study include the fact that BMSCs can also enhance neurological function post-CPR through the secretion of neurotrophic factor [41] and exosome [42]. BMSCs overexpressing brain-derived neurotrophic factor (BDNF) and vascular endothelial growth factor (VEGF) demonstrate substantial therapeutic efficacy in mitigating brain damage following CA [43]. These experiments could not preclude the potential interference of these BMSC-derived factors on mitochondrial homeostasis due to technical constraints. Second, during CPR, all organs can experience ischemia-reperfusion injury, and transplanted BMSCs delivered via the veins may become trapped in the pulmonary circulation. Additionally, BMSCs can ameliorate the effects of ischemia-reperfusion injury on the lungs [44] and liver [45]. It is plausible that BMSCs may initiate a cascading effect through which they restore neurological function by treating other organs. Third, the hippocampus, known for being highly vulnerable to hypoxia/ischemic damage, was selected as the focal region to assess the therapeutic capabilities of transplanted BMSCs. Although the transplantation of BMSCs can mitigate cortical cell pyroptosis following CPR, its influence on mitochondrial homeostasis remains undetermined [46]. Further research is thus required to explore the impact of BMSC transplantation on mitochondrial homeostasis in brain regions outside the hippocampus. Considering the absence of supporting cell-based experiments, our forthcoming studies will illustrate the neuroprotective potential of transplanted BMSCs on rat CPR outcomes using glucose-oxygen deprivation and oxidative stress model systems.

## Conclusion

In summary, the results of this study demonstrate that the neuroprotective mechanisms through which BMSCs function after CPR involve the maintenance of mitochondrial homeostasis and reductions in neuronal apoptosis through the dual regulatory effects of mitophagy and exogenous mitochondrial transfer. The core protein Miro1 optimizes the neuroprotective effects of BMSCs in this context by regulating mitochondrial transfer and autophagy.

## Supplementary Information

Below is the link to the electronic supplementary material.

Supplementary Material 1. In vitro differentiation of BMSCs and mtDNA level.

Supplementary Material 2.

## Data Availability

The datasets generated and analyzed during this study are available from the corresponding author upon reasonable request.

## Abbreviations

CA: Cardiac arrest
CPR: Cardiopulmonary resuscitation
ROSC: Restoration of spontaneous circulation
I/R: Ischemia/reperfusion
ROS: Reactive oxygen species
MSC: Mesenchymal stem cell
BMSCs: Bone marrow mesenchymal stem cell
MMP: Mitochondrial membrane potential
NDS: Neurological Deficit Score
PINK1: PTEN-induced kinase 1
OMM: Outer mitochondrial membrane
BDNF: Brain-derived neurotrophic factor
VEGF: Vascular endothelial growth factor

## References

[1] Myat A, Song K-J, Rea T. Out-of-hospital cardiac arrest: current concepts. Lancet. 2018;391:970–9. 29536861 10.1016/S0140-6736(18)30472-0

[2] Xu F, Zhang Y, Chen Y. Cardiopulmonary resuscitation training in china: current situation and future development. JAMA Cardiol. 2017;2:469–70. 28297007 10.1001/jamacardio.2017.0035

[3] Andersen LW, Holmberg MJ, Berg KM, Donnino MW, Granfeldt A. In-Hospital cardiac arrest: A review. JAMA. 2019;321:1200–10. 30912843 10.1001/jama.2019.1696 PMC6482460

[4] Fugate JE. Anoxic-Ischemic brain injury. Neurol Clin. 2017;35:601–11. 28962803 10.1016/j.ncl.2017.06.001

[5] Pulsinelli WA, Brierley JB, Plum F. Temporal profile of neuronal damage in a model of transient forebrain ischemia. Ann Neurol. 1982;11:491–8. 7103425 10.1002/ana.410110509

[6] Laver S, Farrow C, Turner D, Nolan J. Mode of death after admission to an intensive care unit following cardiac arrest. Intensive Care Med. 2004;30:2126–8. 15365608 10.1007/s00134-004-2425-z

[7] Zhou X, Liu Y, Huang Y, Zhu S, Zhu J, Wang R. Hypertonic saline infusion suppresses apoptosis of hippocampal cells in a rat model of cardiopulmonary resuscitation. Sci Rep. 2017;7:5783. 28724904 10.1038/s41598-017-05919-4 PMC5517425

[8] Zorov DB, Juhaszova M, Sollott SJ. Mitochondrial reactive oxygen species (ROS) and ROS-induced ROS release. Physiol Rev. 2014;94:909–50. 24987008 10.1152/physrev.00026.2013 PMC4101632

[9] Zhou X, Yong L, Huang Y, Zhu S, Song X, Li B, et al. The protective effects of distal ischemic treatment on apoptosis and mitochondrial permeability in the hippocampus after cardiopulmonary resuscitation. J Cell Physiol. 2018;233:6902–10. 29323705 10.1002/jcp.26459 PMC13482420

[10] Lin SR, Lin QM, Lin YJ, Qian X, Wang XP, Gong Z, et al. Bradykinin postconditioning protects rat hippocampal neurons after restoration of spontaneous circulation following cardiac arrest via activation of the AMPK/mTOR signaling pathway. Neural Regen Res. 2022;17:2232–7. 35259843 10.4103/1673-5374.337049 PMC9083139

[11] Leong K-H, Zhou L-L, Lin Q-M, Wang P, Yao L, Huang Z-T. Therapeutic effects of various methods of MSC transplantation on cerebral resuscitation following cardiac arrest in rats. Mol Med Rep. 2016;13:3043–51. 26935023 10.3892/mmr.2016.4927 PMC4805067

[12] Colter DC, Class R, DiGirolamo CM, Prockop DJ. Rapid expansion of recycling stem cells in cultures of plastic-adherent cells from human bone marrow. Proc Natl Acad Sci U S A. 2000;97:3213–8. 10725391 10.1073/pnas.070034097 PMC16218

[13] Jiang D, Gao F, Zhang Y, Wong DSH, Li Q, Tse H-F, et al. Mitochondrial transfer of mesenchymal stem cells effectively protects corneal epithelial cells from mitochondrial damage. Cell Death Dis. 2016;7:e2467. 27831562 10.1038/cddis.2016.358 PMC5260876

[14] Lv M, Zhang S, Jiang B, Cao S, Dong Y, Cao L, et al. Adipose-derived stem cells regulate metabolic homeostasis and delay aging by promoting mitophagy. FASEB J Off Publ Fed Am Soc Exp Biol. 2021;35:e21709. 10.1096/fj.202100332R 34143518

[15] Kontou G, Antonoudiou P, Podpolny M, Szulc BR, Arancibia-Carcamo IL, Higgs NF, et al. Miro1-dependent mitochondrial dynamics in parvalbumin interneurons. eLife. 2021;10:e65215. 34190042 10.7554/eLife.65215 PMC8294849

[16] Safiulina D, Kuum M, Choubey V, Gogichaishvili N, Liiv J, Hickey MA, et al. Miro proteins prime mitochondria for parkin translocation and mitophagy. EMBO J. 2019;38:e99384. 30504269 10.15252/embj.201899384 PMC6331716

[17] Schwarz L, Sharma K, Dodi LD, Rieder L-S, Fallier-Becker P, Casadei N, et al. Miro1 R272Q disrupts mitochondrial calcium handling and neurotransmitter uptake in dopaminergic neurons. Front Mol Neurosci. 2022;15:966209. 36533136 10.3389/fnmol.2022.966209 PMC9757607

[18] Idris AH, Becker LB, Ornato JP, Hedges JR, Bircher NG, Chandra NC, et al. Utstein-style guidelines for uniform reporting of laboratory CPR research. A statement for healthcare professionals from a task force of the American Heart Association, the American College of Emergency Physicians, the American College of Cardiology, the European Resuscitation Council, the Heart and Stroke Foundation of Canada, the Institute of Critical Care Medicine, the Safar Center for Resuscitation Research, and the Society for Academic Emergency Medicine. Writing Group. Circulation. 1996;94:2324–36. 8901707 10.1161/01.cir.94.9.2324

[19] Soleimani M, Nadri S. A protocol for isolation and culture of mesenchymal stem cells from mouse bone marrow. Nat Protoc. 2009;4:102–6. 19131962 10.1038/nprot.2008.221

[20] Dominici M, Le Blanc K, Mueller I, Slaper-Cortenbach I, Marini F, Krause D, et al. Minimal criteria for defining multipotent mesenchymal stromal cells. The international society for cellular therapy position statement. Cytotherapy. 2006;8:315–7. 16923606 10.1080/14653240600855905

[21] Geocadin RG, Ghodadra R, Kimura T, Lei H, Sherman DL, Hanley DF, et al. A novel quantitative EEG injury measure of global cerebral ischemia. Clin Neurophysiol. 2000;111:1779–87. 11018492 10.1016/s1388-2457(00)00379-5

[22] Wang T, Tang W, Sun S, Xu T, Wang H, Guan J, et al. Intravenous infusion of bone marrow mesenchymal stem cells improves brain function after resuscitation from cardiac arrest. Crit Care Med. 2008;36:S486–91. 20449915 10.1097/ccm.0b013e31818a8ff0

[23] Zhou LL, Liang JK, Lin QM, Huang ZT. Effect and mechanism of different ways of transplanting bone marrow mesenchymal stem cells in cardiopulmonary resuscitation in rats. Genet Mol Res GMR. 2014;13:7937–49. 25299109 10.4238/2014.September.29.7

[24] Gazmuri RJ, Radhakrishnan J. Protecting mitochondrial bioenergetic function during resuscitation from cardiac arrest. Crit Care Clin. 2012;28:245–70. 22433486 10.1016/j.ccc.2012.02.001 PMC3310365

[25] Huang P-J, Kuo C-C, Lee H-C, Shen C-I, Cheng F-C, Wu S-F, et al. Transferring xenogenic mitochondria provides neural protection against ischemic stress in ischemic rat brains. Cell Transpl. 2016;25:913–27. 10.3727/096368915X689785 26555763

[26] Paliwal S, Chaudhuri R, Agrawal A, Mohanty S. Regenerative abilities of mesenchymal stem cells through mitochondrial transfer. J Biomed Sci. 2018;25:31. 29602309 10.1186/s12929-018-0429-1 PMC5877369

[27] Liu K, Guo L, Zhou Z, Pan M, Yan C. Mesenchymal stem cells transfer mitochondria into cerebral microvasculature and promote recovery from ischemic stroke. Microvasc Res. 2019;123:74–80. 30611747 10.1016/j.mvr.2019.01.001

[28] Yang Y, Ye G, Zhang Y-L, He H-W, Yu B-Q, Hong Y-M, et al. Transfer of mitochondria from mesenchymal stem cells derived from induced pluripotent stem cells attenuates hypoxia-ischemia-induced mitochondrial dysfunction in PC12 cells. Neural Regen Res. 2020;15:464–72. 31571658 10.4103/1673-5374.266058 PMC6921344

[29] Chen C, Lu L, Zhu J, Gu X, Liu B, Li D, et al. Miro1 provides neuroprotection via the mitochondrial trafficking pathway in a rat model of traumatic brain injury. Brain Res. 2021;1773:147685. 34637761 10.1016/j.brainres.2021.147685

[30] Panda SP, Prasanth D, Gorla US, Dewanjee S. Interlinked role of ASN, TDP-43 and Miro1 with parkinopathy: focus on targeted approach against neuropathy in parkinsonism. Ageing Res Rev. 2023;83:101783. 36371014 10.1016/j.arr.2022.101783

[31] Wu X, Li X, Liu Y, Yuan N, Li C, Kang Z, et al. Hydrogen exerts neuroprotective effects on OGD/R damaged neurons in rat hippocampal by protecting mitochondrial function via regulating mitophagy mediated by PINK1/Parkin signaling pathway. Brain Res. 2018;1698:89–98. 29958907 10.1016/j.brainres.2018.06.028

[32] Guan R, Zou W, Dai X, Yu X, Liu H, Chen Q, et al. Mitophagy, a potential therapeutic target for stroke. J Biomed Sci. 2018;25:87. 30501621 10.1186/s12929-018-0487-4 PMC6271612

[33] Zeng K, Yu X, Mahaman YA, Wang JZ, Liu R, Li Y, et al. Defective mitophagy and the etiopathogenesis of alzheimer’s disease. Transl Neurodegener. 2022;11:32. 35655270 10.1186/s40035-022-00305-1 PMC9164340

[34] Silvian LF. PINK1/Parkin pathway activation for mitochondrial quality control - which is the best molecular target for therapy? Front Aging Neurosci. 2022;14:890823. 35754955 10.3389/fnagi.2022.890823 PMC9215347

[35] Li S, Sheng Z-H. Energy matters: presynaptic metabolism and the maintenance of synaptic transmission. Nat Rev Neurosci. 2022;23:4–22. 34782781 10.1038/s41583-021-00535-8

[36] Ahmad M, Dar NJ, Bhat ZS, Hussain A, Shah A, Liu H, et al. Inflammation in ischemic stroke: mechanisms, consequences and possible drug targets. CNS Neurol Disord Drug Targets. 2014;13:1378–96. 25345517 10.2174/1871527313666141023094720

[37] Mills EL, Kelly B, Logan A, Costa ASH, Varma M, Bryant CE, et al. Succinate dehydrogenase supports metabolic repurposing of mitochondria to drive inflammatory macrophages. Cell. 2016;167:457–e47013. 27667687 10.1016/j.cell.2016.08.064 PMC5863951

[38] Cirotti C, Rizza S, Giglio P, Poerio N, Allega MF, Claps G, et al. Redox activation of ATM enhances GSNOR translation to sustain mitophagy and tolerance to oxidative stress. EMBO Rep. 2021;22:e50500. 33245190 10.15252/embr.202050500 PMC7788447

[39] Balaban RS, Nemoto S, Finkel T. Mitochondria, oxidants, and aging. Cell. 2005;120:483–95. 15734681 10.1016/j.cell.2005.02.001

[40] Li W, Qu Z, Prakash R, Chung C, Ma H, Hoda MN, et al. Comparative analysis of the neurovascular injury and functional outcomes in experimental stroke models in diabetic Goto-Kakizaki rats. Brain Res. 2013;1541:106–14. 24144674 10.1016/j.brainres.2013.10.021 PMC3878856

[41] Tang X, Chen F, Lin Q, You Y, Ke J, Zhao S. Bone marrow mesenchymal stem cells repair the hippocampal neurons and increase the expression of IGF–1 after cardiac arrest in rats. Exp Ther Med. 2017;14:4312–20. 29067112 10.3892/etm.2017.5059 PMC5647699

[42] Li F, Zhang J, Chen A, Liao R, Duan Y, Xu Y, et al. Combined transplantation of neural stem cells and bone marrow mesenchymal stem cells promotes neuronal cell survival to alleviate brain damage after cardiac arrest via microRNA-133b incorporated in extracellular vesicles. Aging. 2021;13:262–78. 33436530 10.18632/aging.103920 PMC7835040

[43] Zhou L, Lin Q, Wang P, Yao L, Leong K, Tan Z, et al. Enhanced neuroprotective efficacy of bone marrow mesenchymal stem cells co-overexpressing BDNF and VEGF in a rat model of cardiac arrest-induced global cerebral ischemia. Cell Death Dis. 2017;8:e2774. 28492549 10.1038/cddis.2017.184 PMC5520708

[44] Li J, Zhou J, Zhang D, Song Y, She J, Bai C. Bone marrow-derived mesenchymal stem cells enhance autophagy via PI3K/AKT signalling to reduce the severity of ischaemia/reperfusion-induced lung injury. J Cell Mol Med. 2015;19:2341–51. 26177266 10.1111/jcmm.12638 PMC4594676

[45] Zheng J, Chen L, Lu T, Zhang Y, Sui X, Li Y, et al. MSCs ameliorate hepatocellular apoptosis mediated by PINK1-dependent mitophagy in liver ischemia/reperfusion injury through AMPKα activation. Cell Death Dis. 2020;11:256. 32312955 10.1038/s41419-020-2424-1 PMC7171190

[46] Tang X, Ke J, Chen F, Lin Q, You Y, Zheng N, et al. Hypoxic preconditioned mesenchymal stem cells ameliorate rat brain injury after cardiopulmonary resuscitation by suppressing neuronal pyroptosis. J Cell Mol Med. 2023;27:1836–58. 37246833 10.1111/jcmm.17782 PMC10315812

