# Supplementary material for: Miro1 protects against brain injury after CPR in rats by enhancing the effect of BMSCs on mitochondrial homeostasis
Source: Stem Cell Res Ther. 2025 Oct 28;16:585. doi: 10.1186/s13287-025-04724-5 (PMC12570573; doi:10.1186/s13287-025-04724-5)
Supplement: Supplementary file 1 — Supplementary Material 1. In vitro differentiation of BMSCs and mtDNA level. [file 13287_2025_4724_MOESM1_ESM.docx]

**Supplement material 1**

**In vitro differentiation of BMSCs**

**Methods：Osteogenic differentiation:** To induce osteogenic differentiation, a specialized medium designed for this purpose was used on rat bone marrow mesenchymal stem cells(PD-008, Procell). BMSCs were seeded into 6-well plates (2×10⁴ cells/well) and treated with various agents. At 90% confluence, cultures were switched to osteogenic induction medium (rat BMSCs formulation) with medium refreshment every 3 days for 15 days. Calcium deposition was assessed by alizarin red staining.

**Result:** Cells differentiated into mineralizing cells stained with alizarin red.
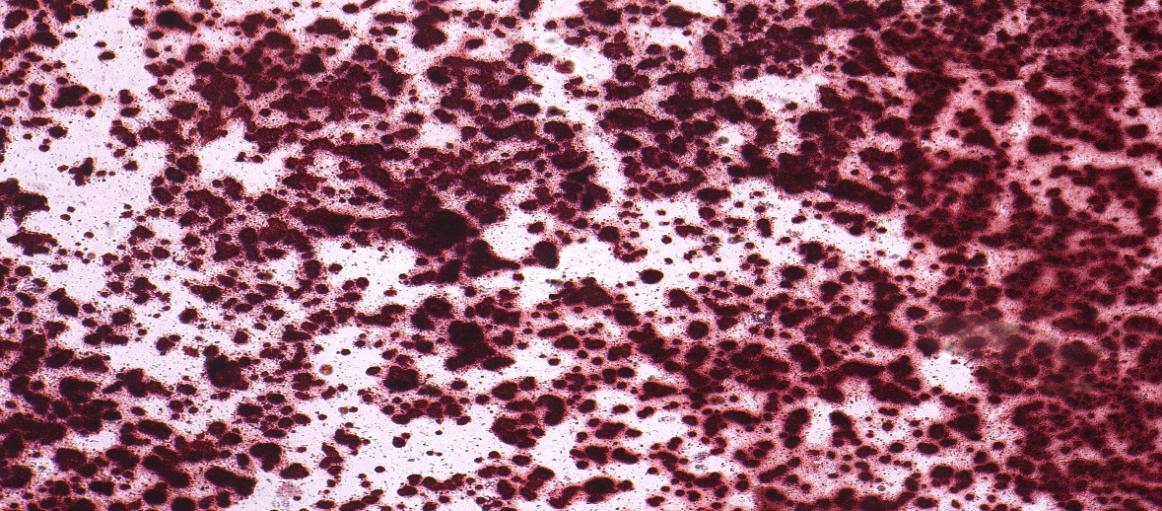


Fig. 1 Cells differentiated into mineralizing cells stained with alizarin red

**The mtDNA level**

**Methods：**Total RNA was extracted from the cells in each group using TRIzol (G3013, Servicebio), and the concentration of the total RNA was determined by measuring the OD260/OD280 ratio with an ultramicrospectrophotometer. Following the manufacturer’s instructions, the Revert Aid First Strand cDNA synthesis kit was utilized for cDNA synthesis. Each qPCR reaction mixture included a total volume of 20 μL, containing 5 μL of DNA template, 0.5 μL of forward primer (10 μmol/L), 0.5 μL of reverse primer (10 μmol/L), 10 μL of 2× SYBR Green qPCR SuperMix, and 4 μL of Nuclease-Free Water. The amplification reaction consisted of 95°C for 5 minutes followed by 40 cycles of denaturation at 95°C for 30 seconds, annealing at 60°C for 30 seconds, and extension at 72°C for 30 seconds. The relative expression levels of genes in each group were calculated using 2-ΔΔCt with Gapdh as the internal reference.

The mtDNA was probed using primers for the specific detection of Complex II (succinate-ubiquinone oxidoreductase): forward, 5′- TACCCGGACTGGATTCTACG -3′, and reverse, 5′- AAGTTGGTGGGCTGTCAATC -3′. For normalization, the following GAPDH primers were used: forward, 5′- AGACAGCCGCATCTTCTTGT -3′, and reverse, 5′- CTTGCCGTGGGTAGAGTCAT -3′.

**Result:** Through the evaluation of mt DNA content in the hippocampus, we observed a substantial elevation in mtDNA content within the CPR-BMSCs group.


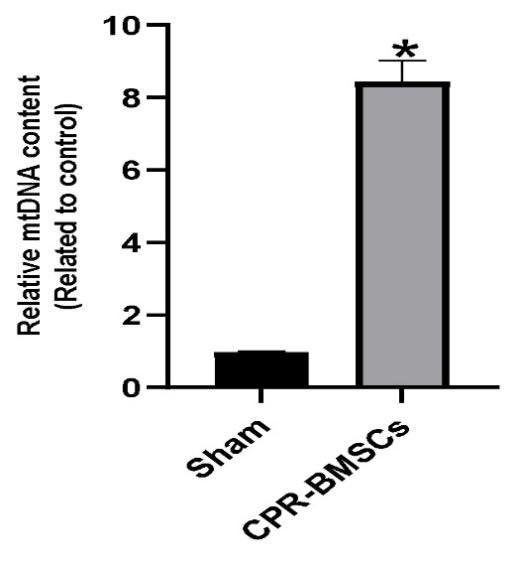


Fig. 2 the relative mtDNA content between the Sham group and the CPR-BMSCs group**P* < 0.05 vs the Sham group
