# Supplementary material for: Miro1 protects against brain injury after CPR in rats by enhancing the effect of BMSCs on mitochondrial homeostasis
Source: Stem Cell Res Ther. 2025 Oct 28;16:585. doi: 10.1186/s13287-025-04724-5 (PMC12570573; doi:10.1186/s13287-025-04724-5)
Supplement: Supplementary file 2 — Supplementary Material 2. [file 13287_2025_4724_MOESM2_ESM.pdf]

Fig 2 d miro1

kDa BMSCs BMSCs-miro1^{v1} BMSCs-miro1^{hi}

180
130
100
72
55
43
37
17

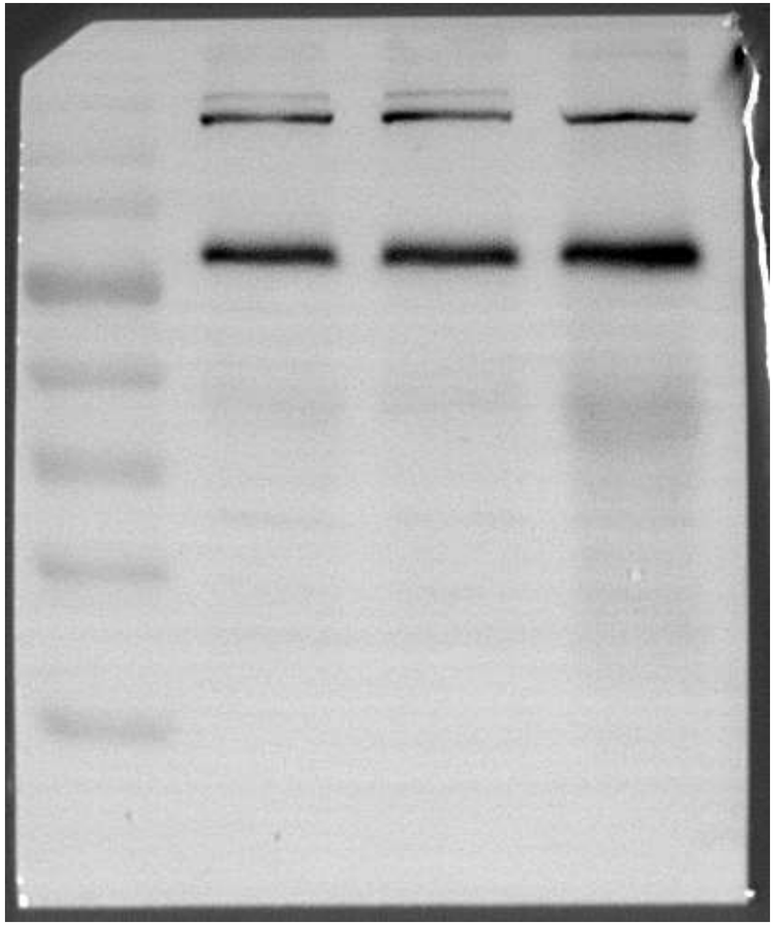

β-actin

kDa BMSCs BMSCs-miro1^{v1} BMSCs-miro1^{hi}

180
130
100
72
55
43
37
17

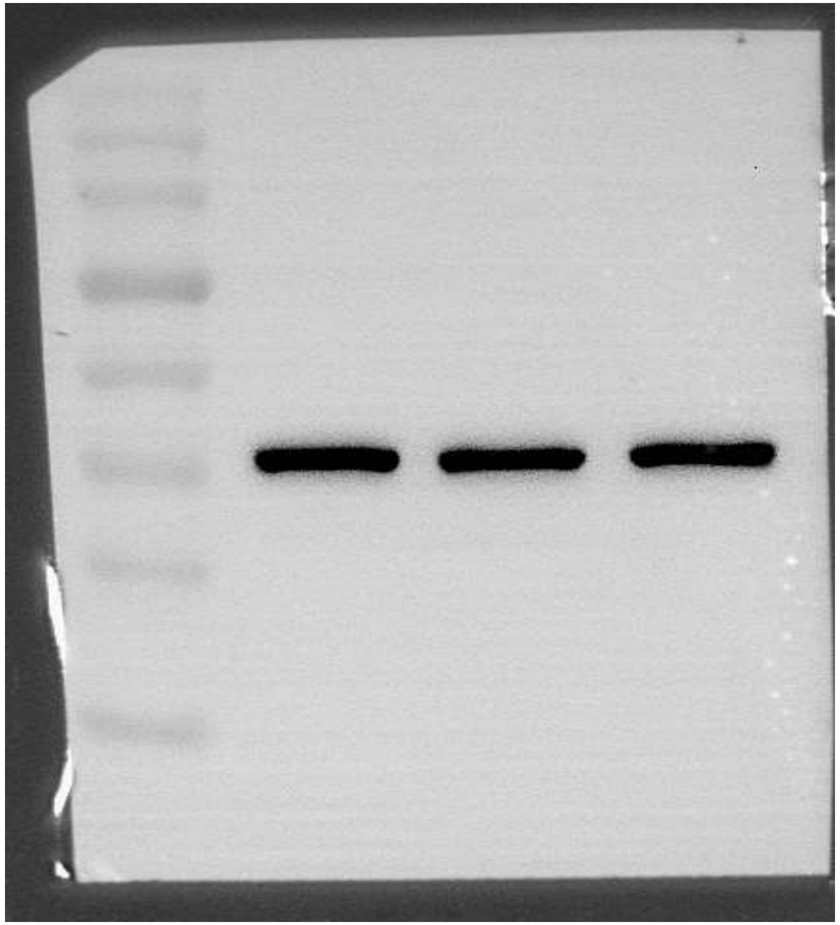

Fig 2 d miro1

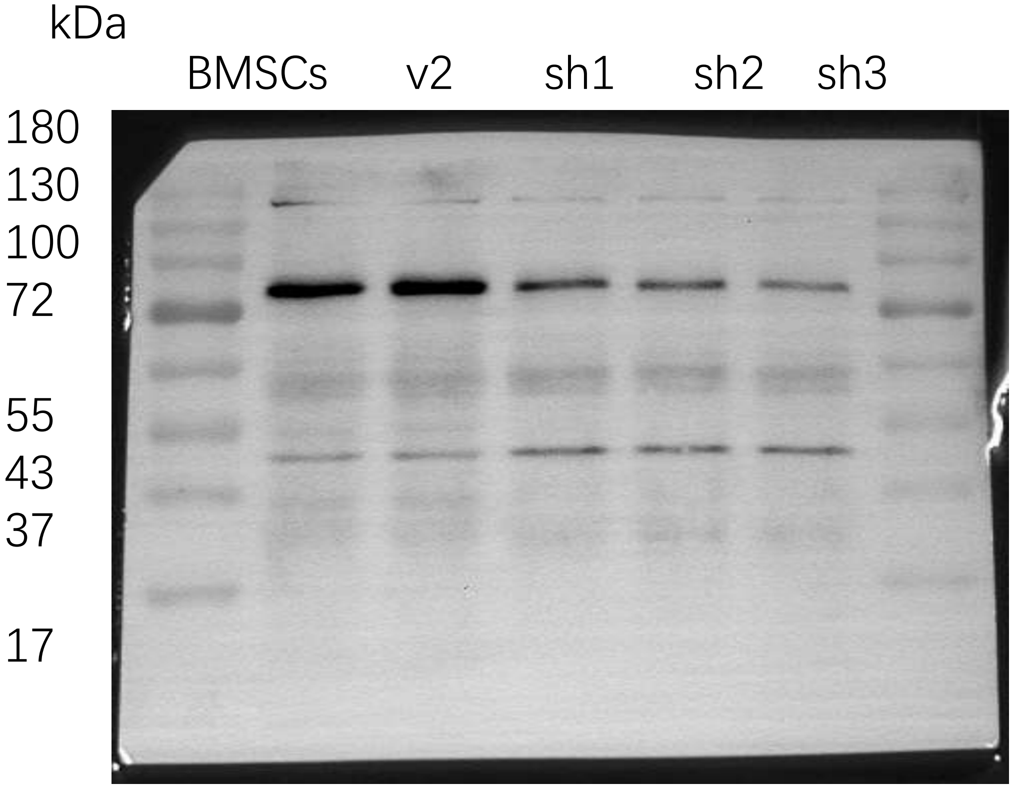

β-actin

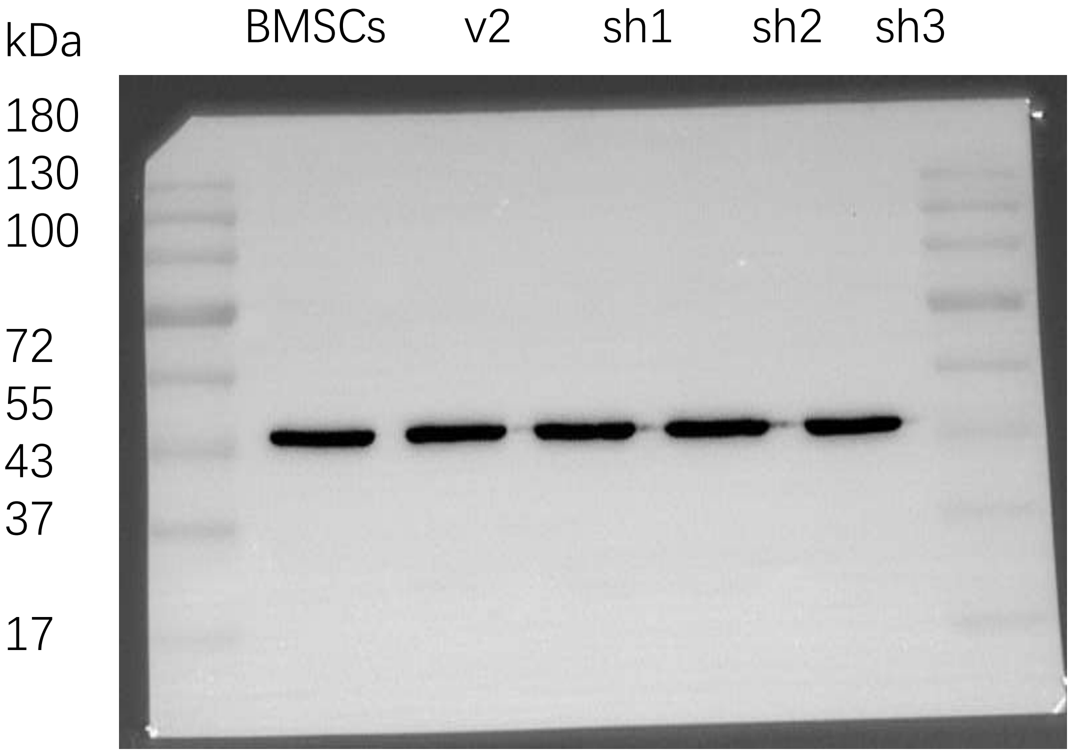

Fig 3 d lc3

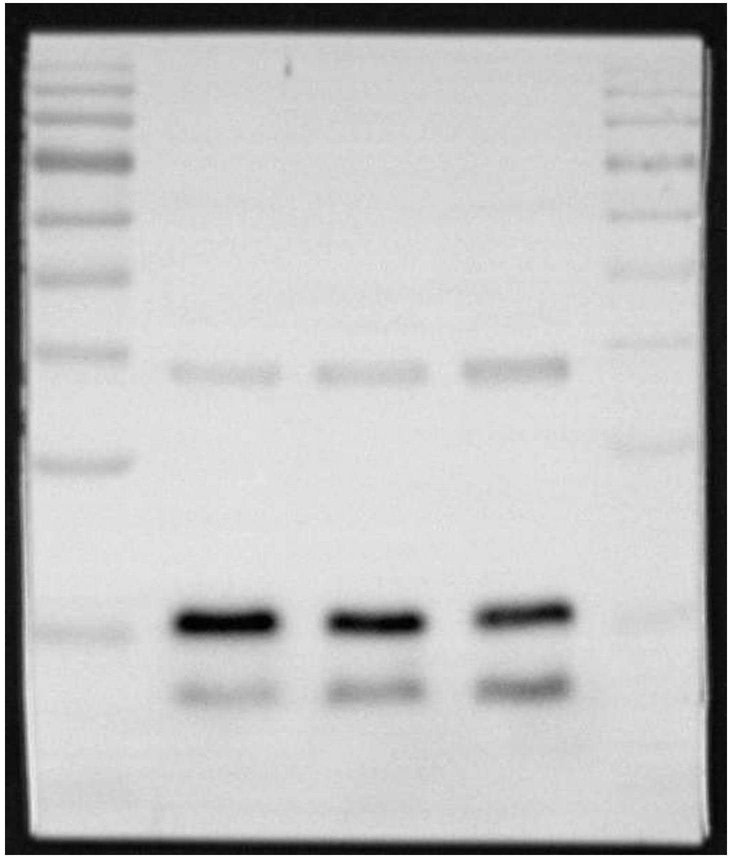

β-actin

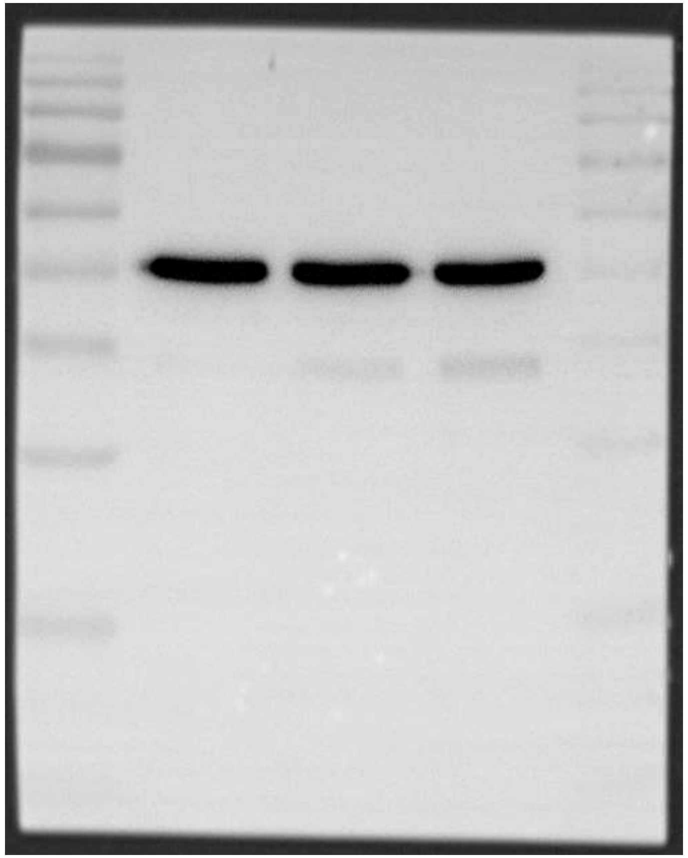

Fig 3 d p62

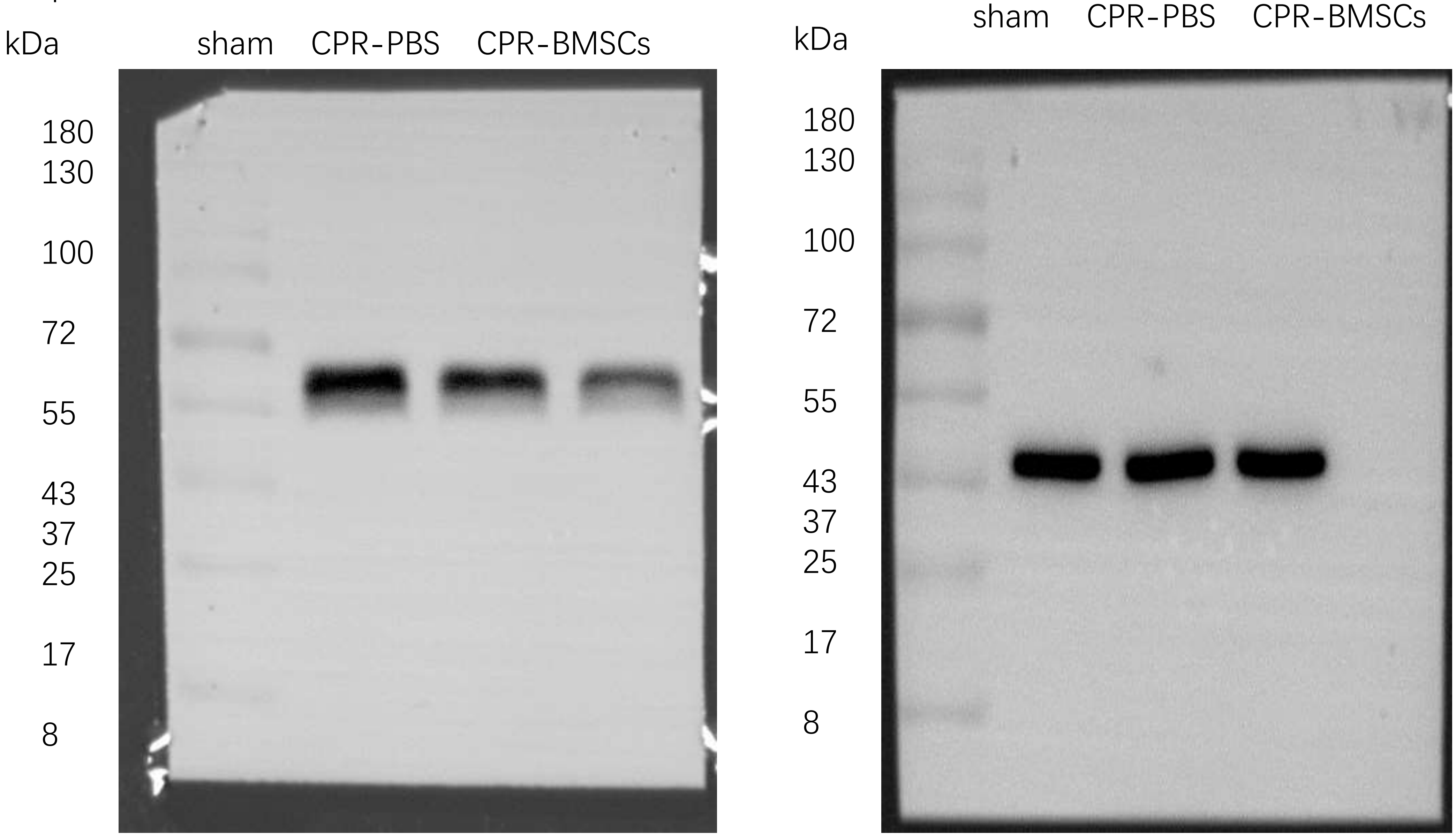
β-actin
kDa
sham
CPR-PBS
CPR-BMSCs
180
130
100
72
55
43
37
25
17
8
kDa
sham
CPR-PBS
CPR-BMSCs
180
130
100
72
55
43
37
25
17
8

Fig 3 d parkin

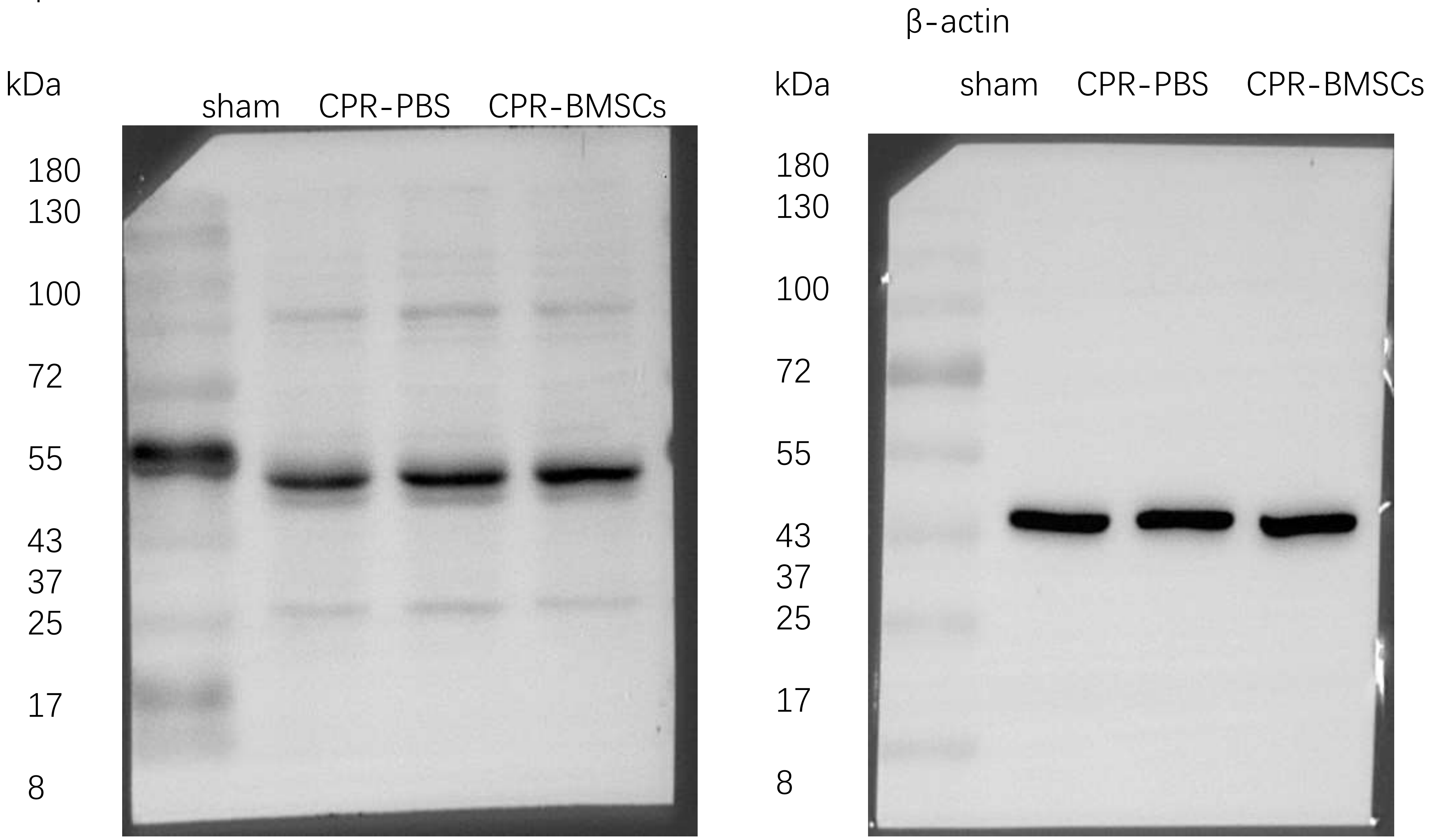

Fig 3 d pink1

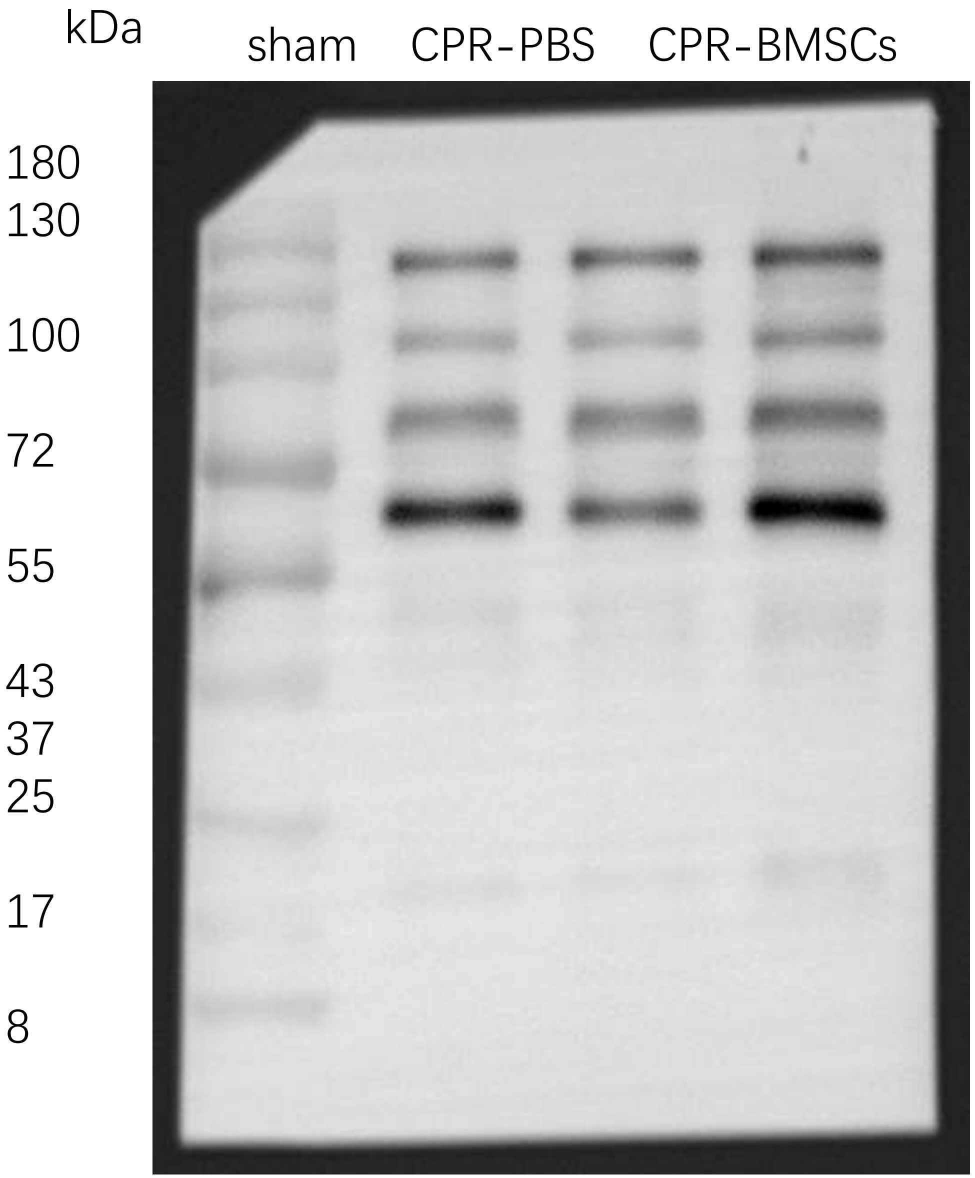

β-actin

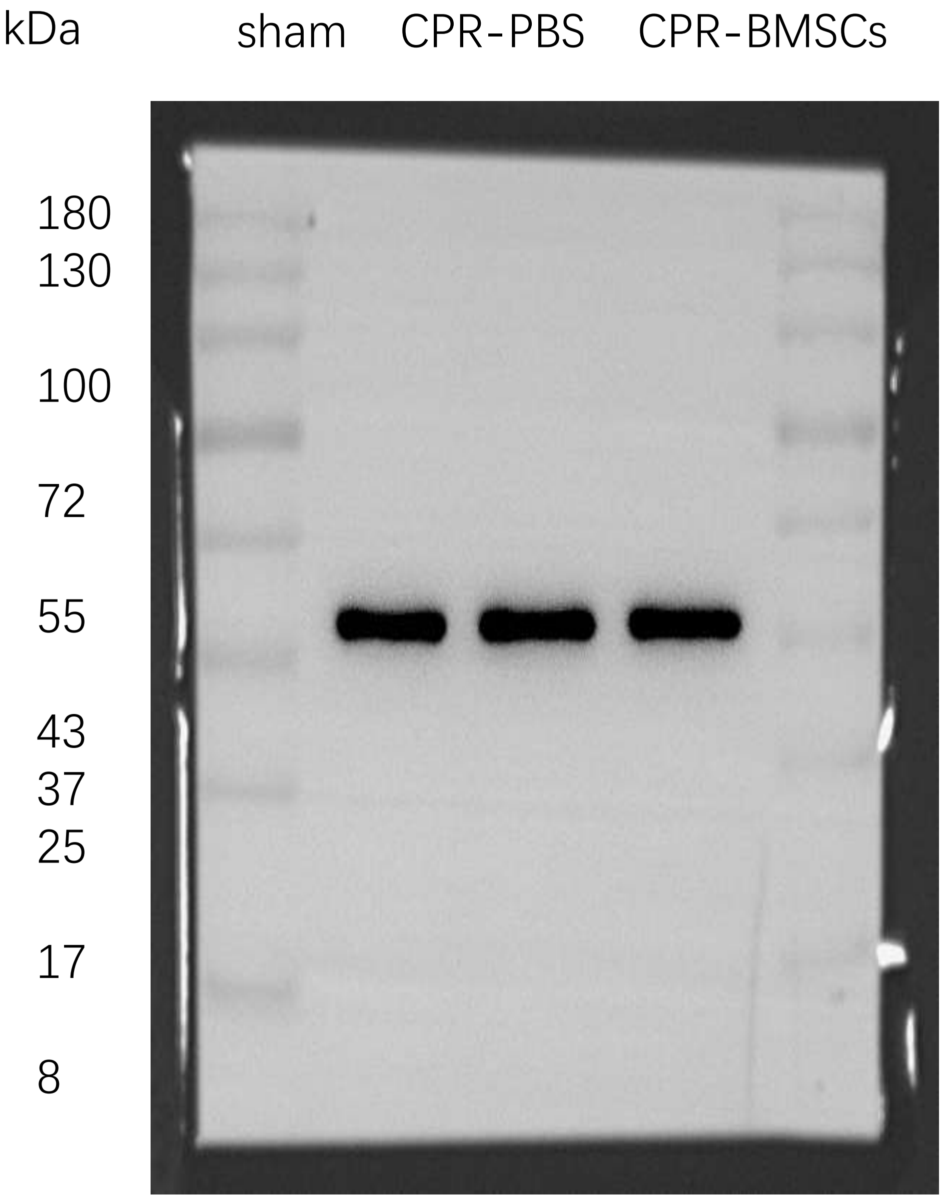

Fig 5 F Tomm20

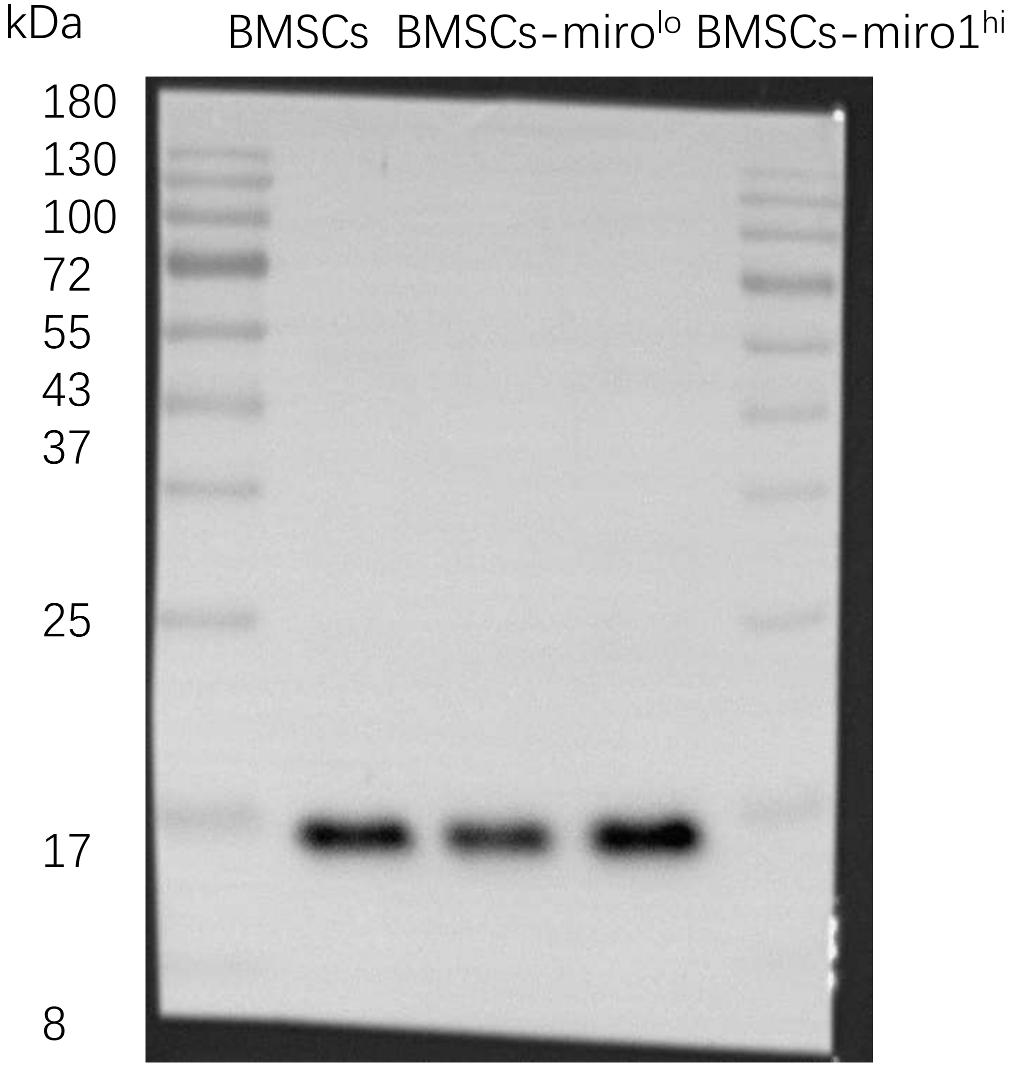

β-actin

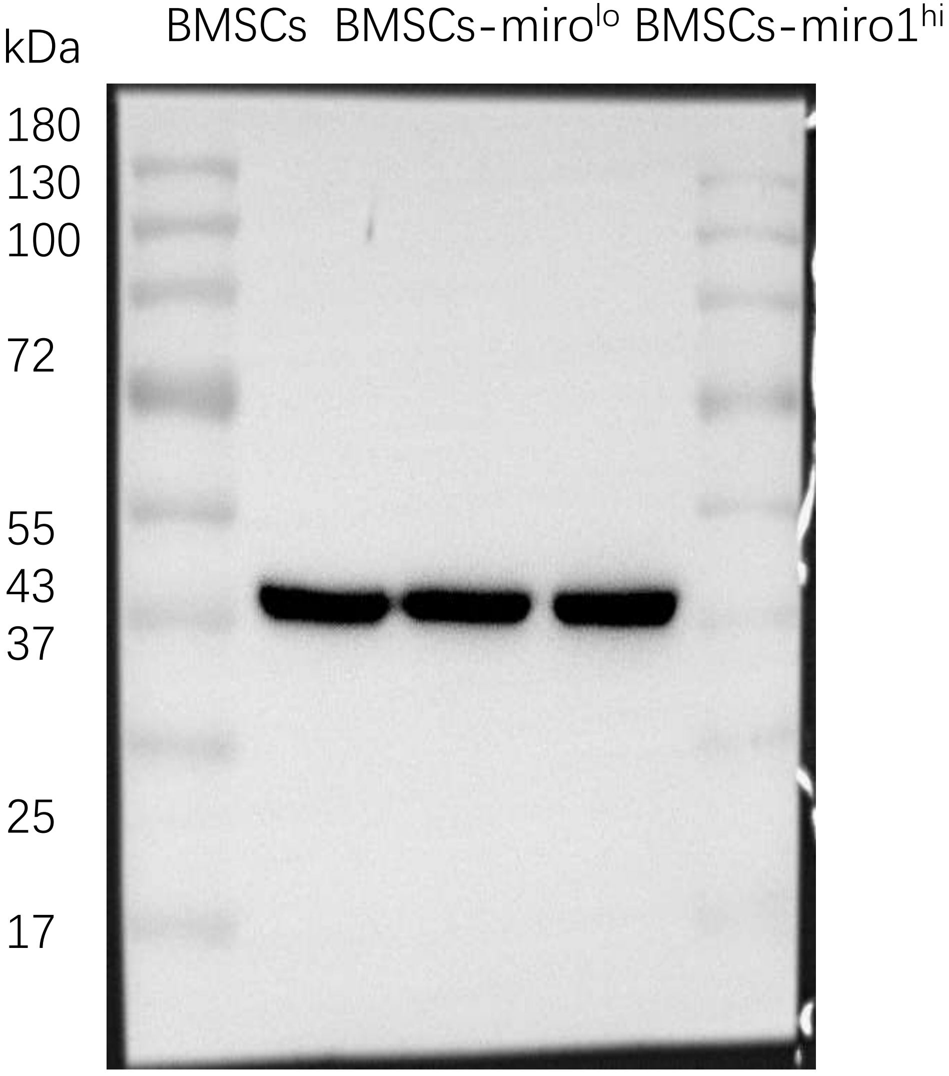

Fig 5 F lc3

kDa

BMSCs BMSCs-miro^{lo} BMSCs-miro1^{hi}

180
130
100
72
55
43
37
25
17
8

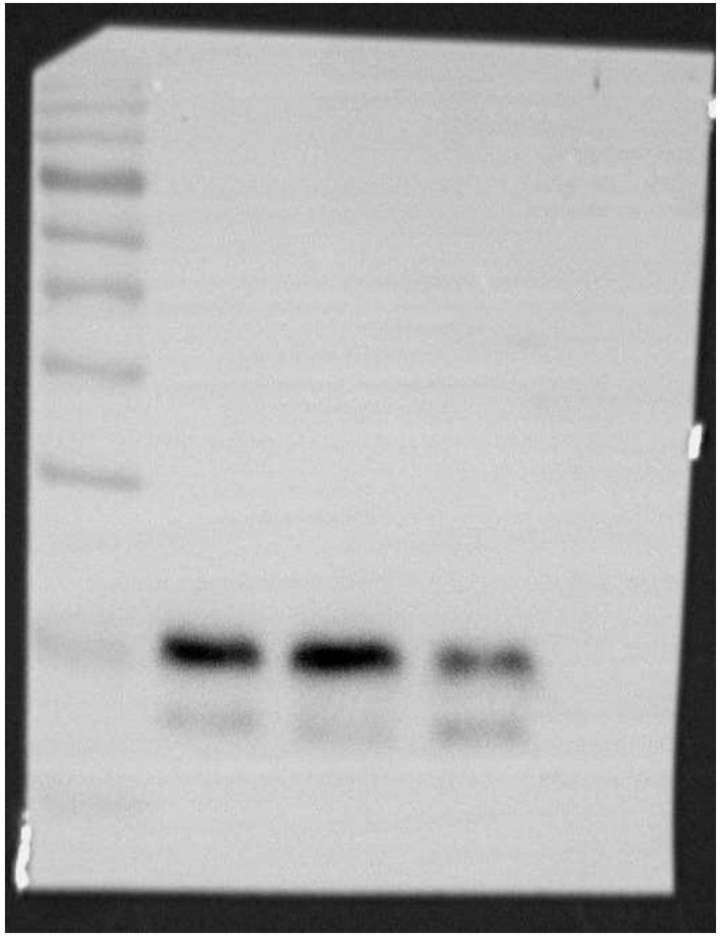

β-actin

kDa BMSCs BMSCs-miro^{lo} BMSCs-miro1^{hi}

180
130
100
72
55
43
37
25
17
8

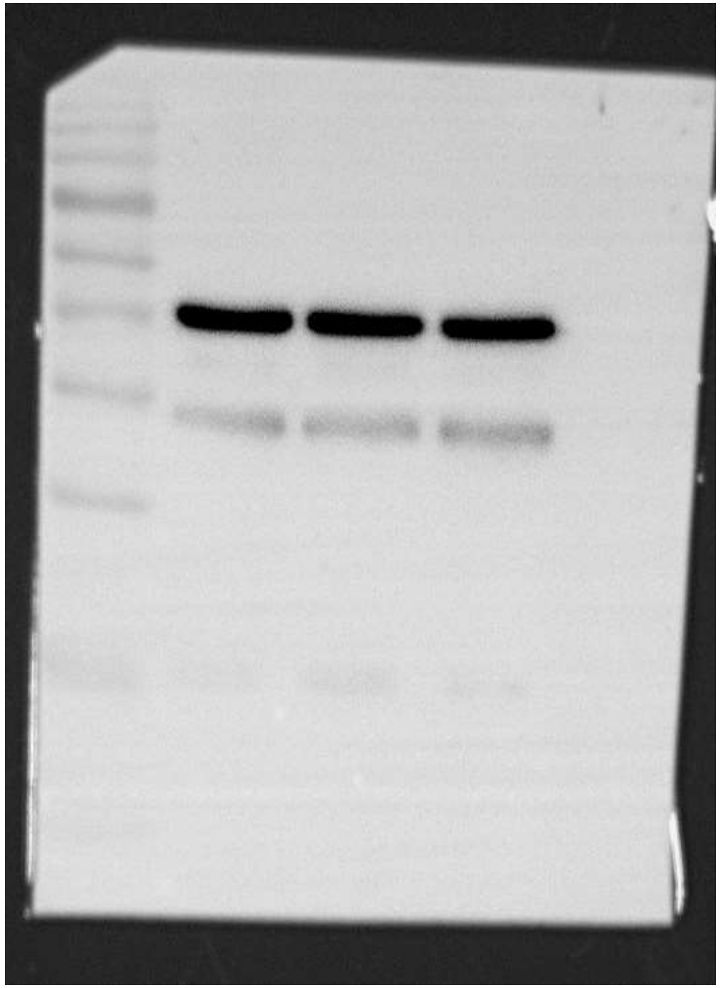

Fig 5 F ATG5

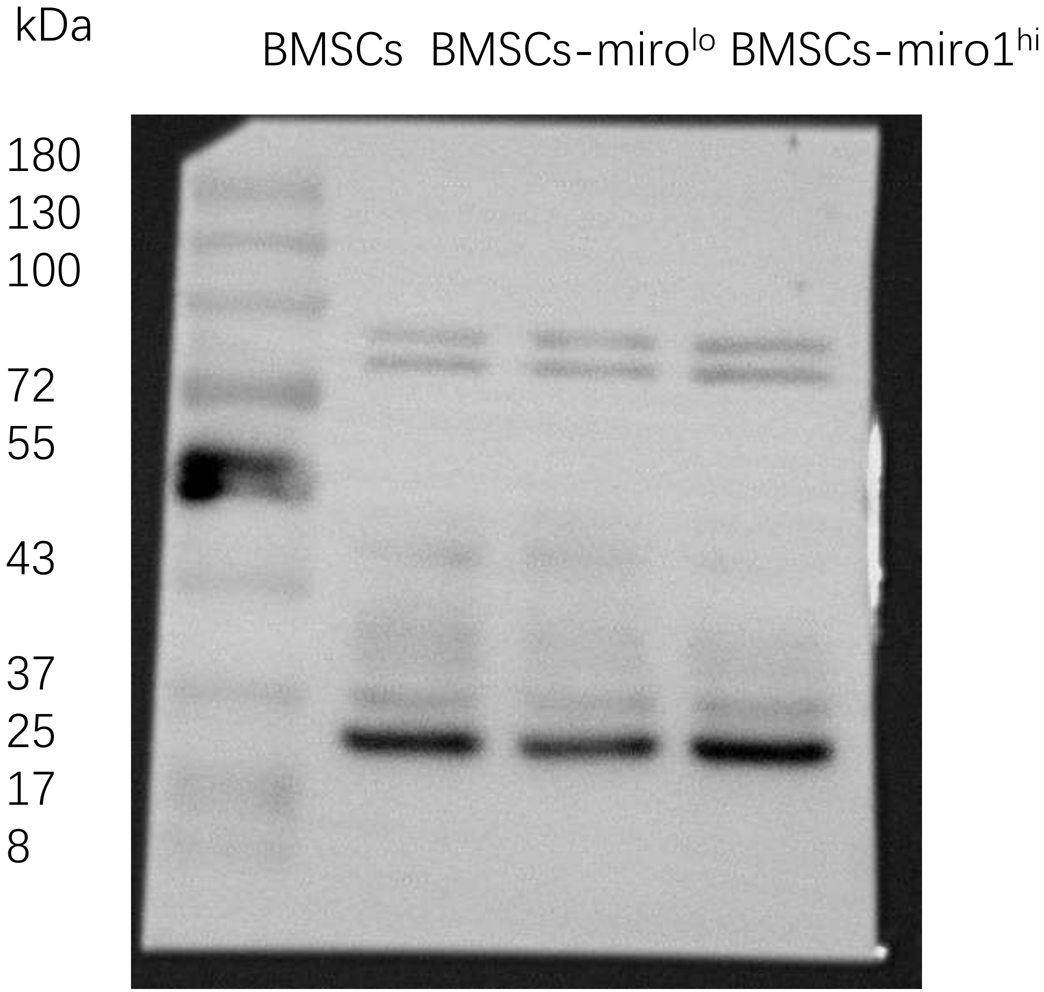

β-actin

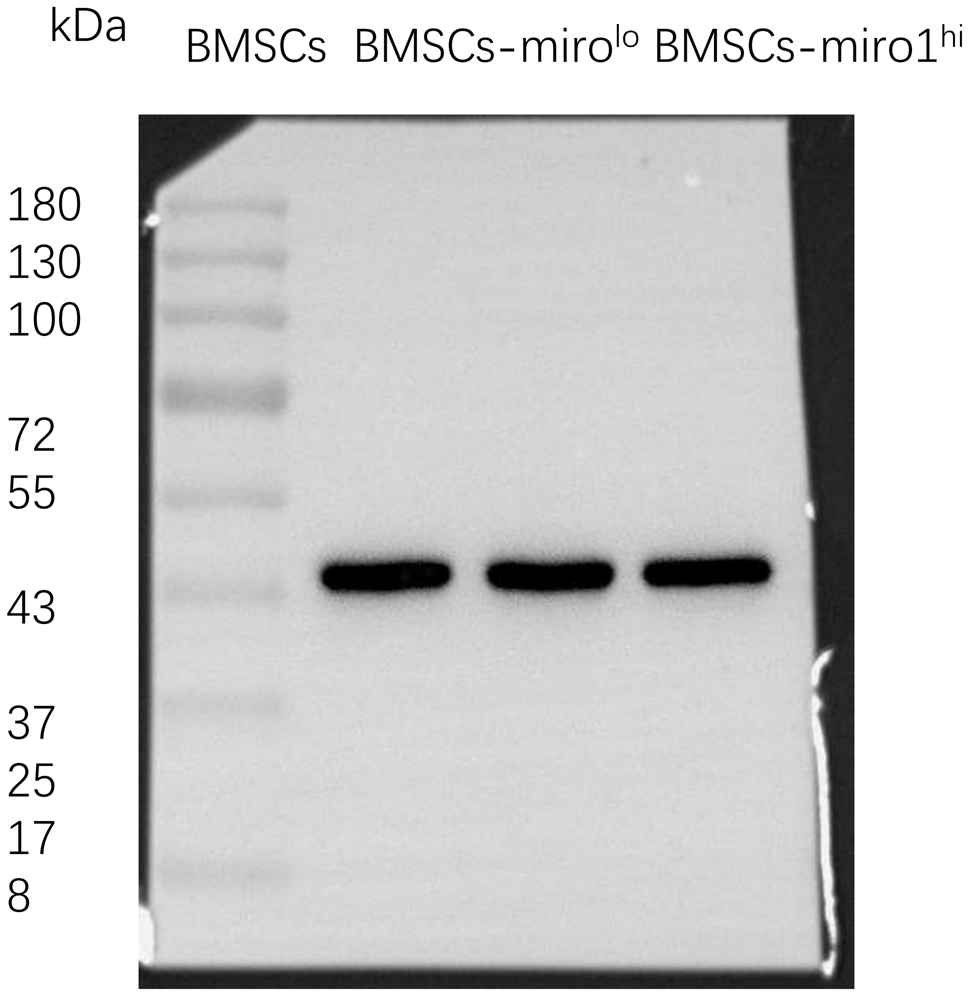

Fig 5 F PINK1

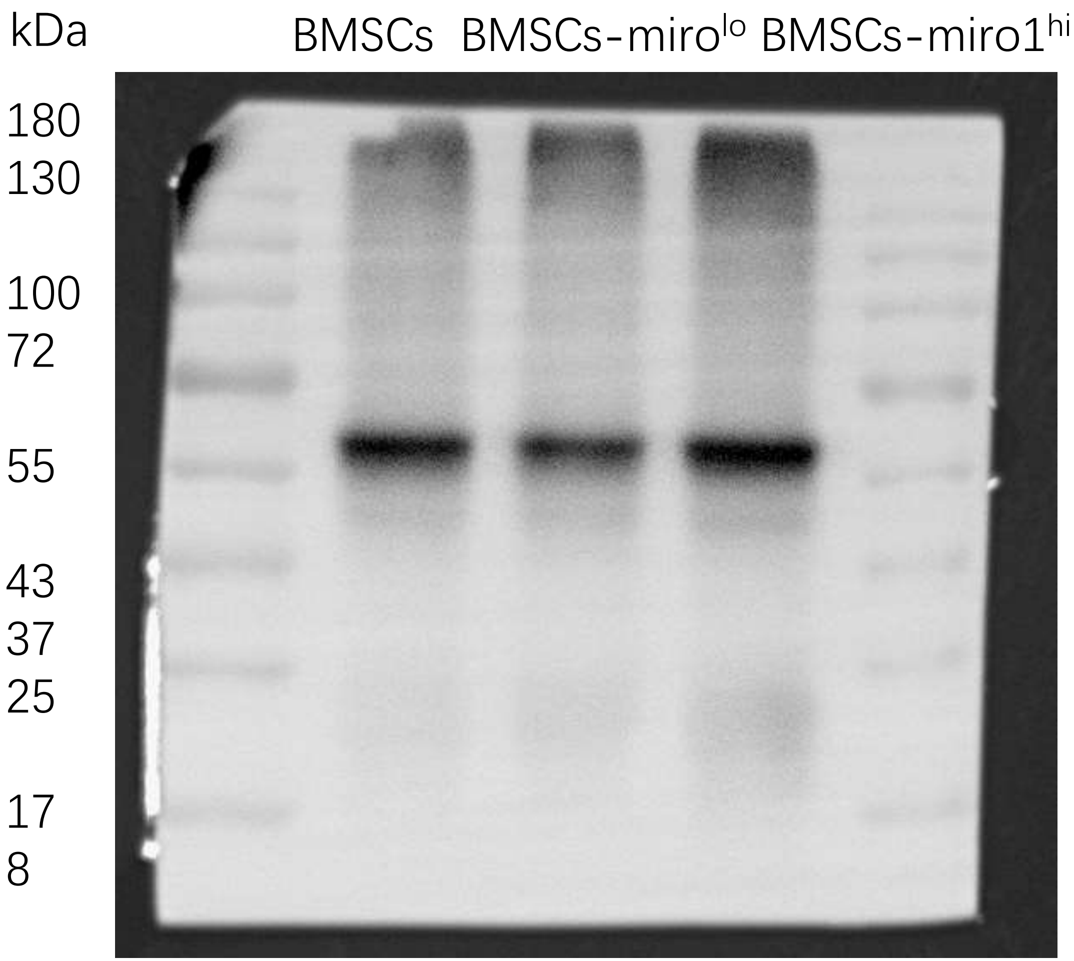

β-actin

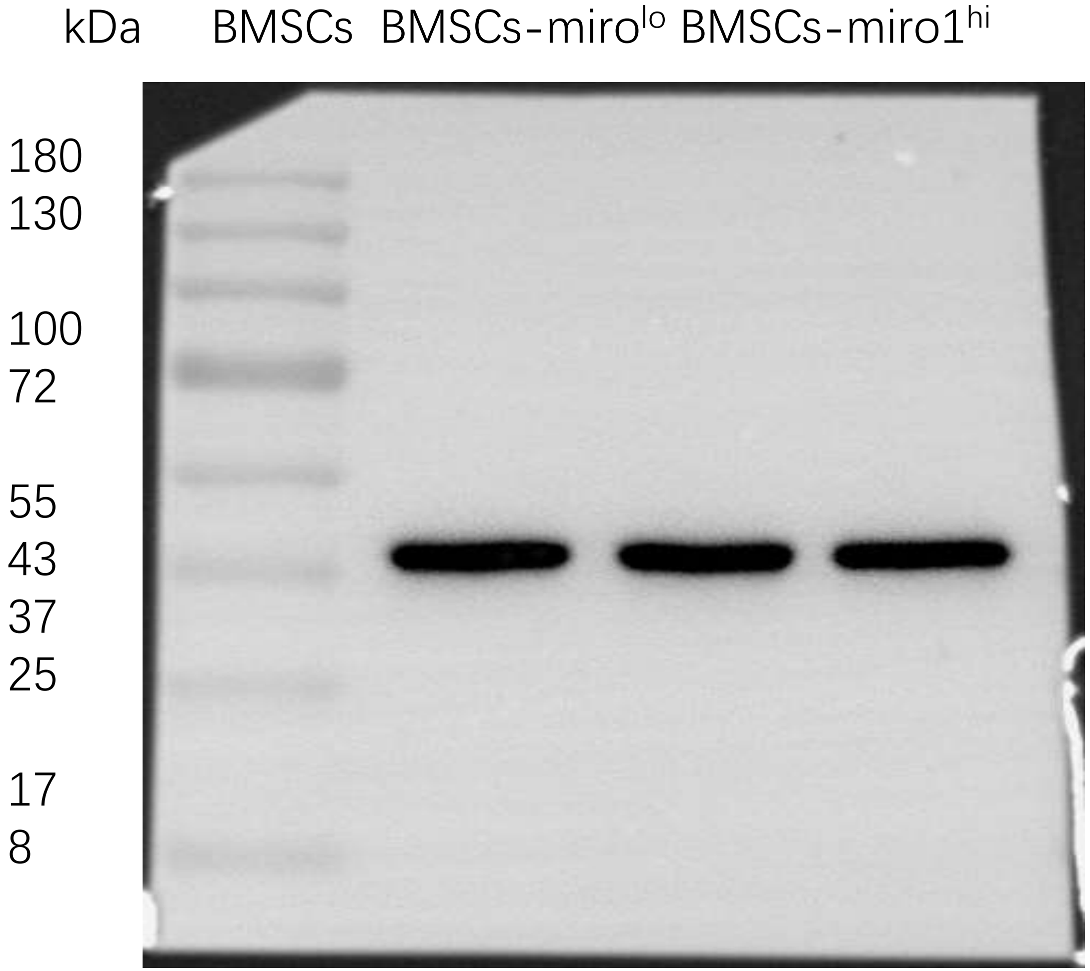

Fig 5 F P62

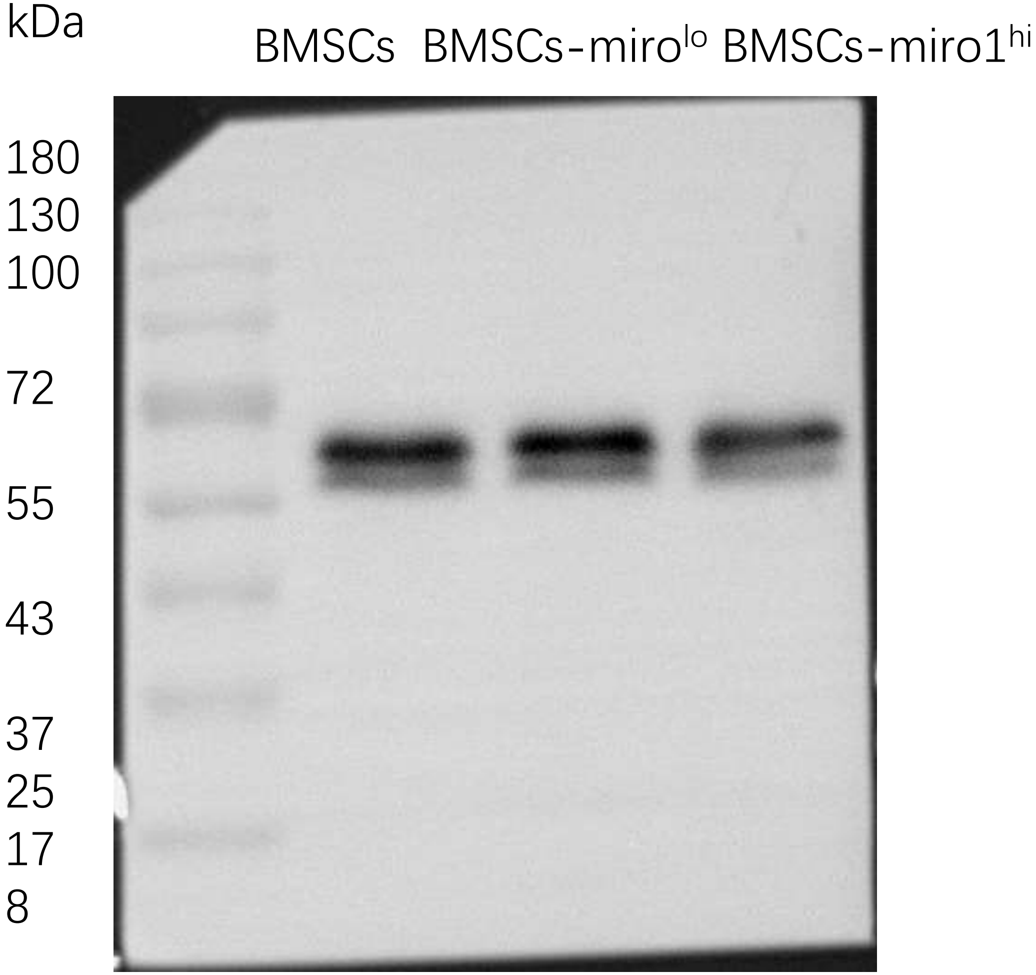

Fig 5 F PARKIN

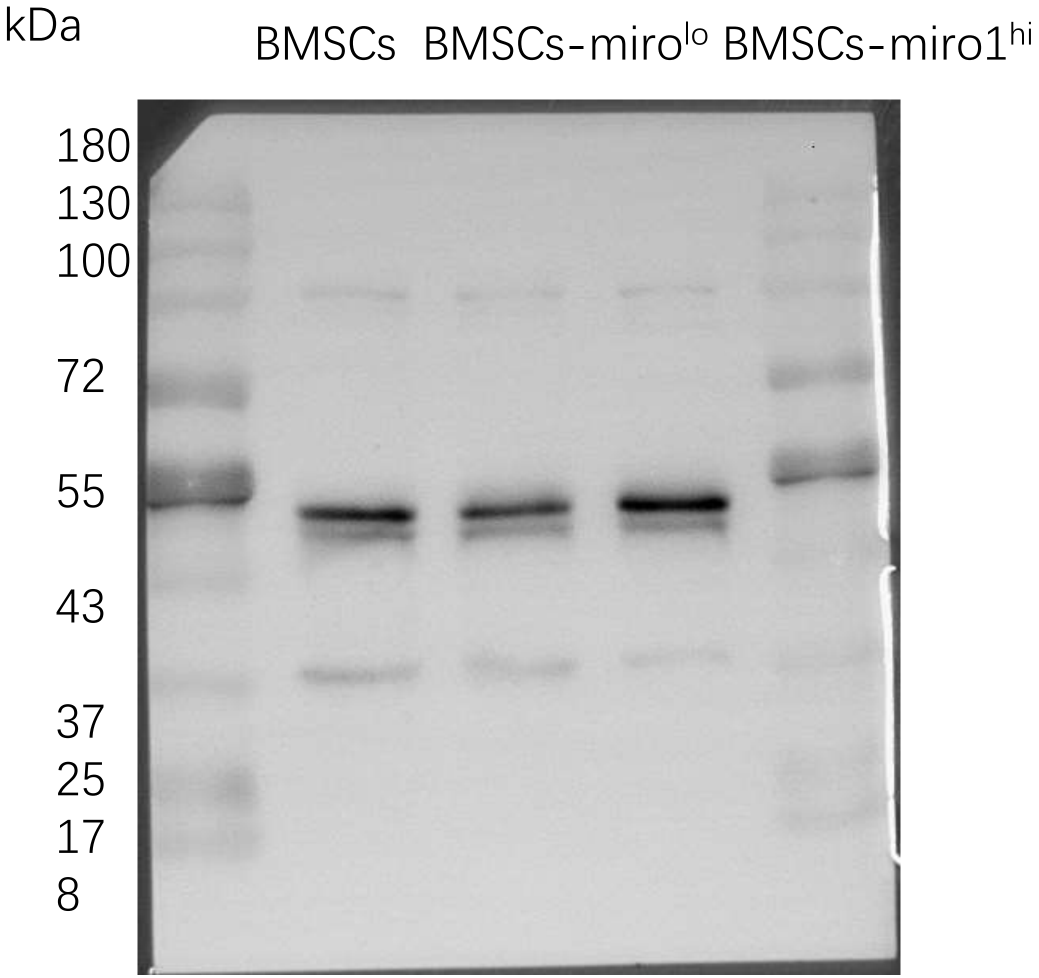

Fig 5 F Mior1

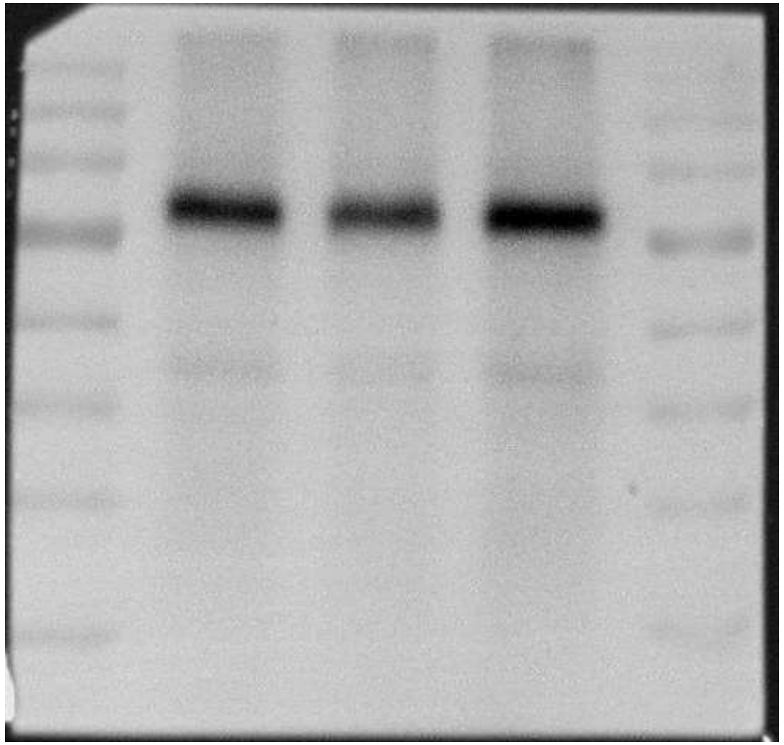
kDa
BMSCs
BMSCs-miro^lo
BMSCs-miro1^hi
180
130
100
72
55
43
37
25
17
8
